# Evolution of the Karyopherin-β Family of Nucleocytoplasmic Transport Factors; Ancient Origins and Continued Specialization

**DOI:** 10.1371/journal.pone.0019308

**Published:** 2011-04-27

**Authors:** Amanda J. O'Reilly, Joel B. Dacks, Mark C. Field

**Affiliations:** 1 Department of Pathology, University of Cambridge, Tennis Court Road, Cambridge, United Kingdom; 2 Department of Cell Biology, University of Alberta, Edmonton, Canada; Texas A&M University, United States of America

## Abstract

**Background:**

Macromolecular transport across the nuclear envelope (NE) is achieved through nuclear pore complexes (NPCs) and requires karyopherin-βs (KAP-βs), a family of soluble receptors, for recognition of embedded transport signals within cargo. We recently demonstrated, through proteomic analysis of trypanosomes, that NPC architecture is likely highly conserved across the Eukaryota, which in turn suggests conservation of the transport mechanisms. To determine if KAP-β diversity was similarly established early in eukaryotic evolution or if it was subsequently layered onto a conserved NPC, we chose to identify KAP-β sequences in a diverse range of eukaryotes and to investigate their evolutionary history.

**Results:**

Thirty six predicted proteomes were scanned for candidate KAP-β family members. These resulting sequences were resolved into fifteen KAP-β subfamilies which, due to broad supergroup representation, were most likely represented in the last eukaryotic common ancestor (LECA). Candidate members of each KAP-β subfamily were found in all eukaryotic supergroups, except XPO6, which is absent from Archaeplastida. Phylogenetic reconstruction revealed the likely evolutionary relationships between these different subfamilies. Many species contain more than one representative of each KAP-β subfamily; many duplications are apparently taxon-specific but others result from duplications occurring earlier in eukaryotic history.

**Conclusions:**

At least fifteen KAP-β subfamilies were established early in eukaryote evolution and likely before the LECA. In addition we identified expansions at multiple stages within eukaryote evolution, including a multicellular plant-specific KAP-β, together with frequent secondary losses. Taken with evidence for early establishment of NPC architecture, these data demonstrate that multiple pathways for nucleocytoplasmic transport were established prior to the radiation of modern eukaryotes but that selective pressure continues to sculpt the KAP-β family.

## Introduction

The major defining feature of eukaryotic cells is the presence of a nucleus, the organelle that sequesters the genetic material away from the cytoplasm. This fundamental cellular architectural modification serves to compartmentalise transcription and translation and likely permitted the evolution of more complex mechanisms for regulating gene expression [1]. Most eukaryotic cells possess additional membrane-bound organelles responsible for secretory and endocytic pathways that almost certainly have endogenous origins; collectively these are referred to as the endomembrane system. Compelling evidence suggests that these structures populated the last eukaryotic common ancestor (LECA) prior to the radiation of modern lineages [2]. It has recently become recognised that there are deep evolutionary relationships between the proteins that deform endomembrane compartments and those serving the nucleus [3] [4].

Trafficking of macromolecules across the nuclear envelope (NE) occurs exclusively through the nuclear pore complex (NPC), a ∼100 MDa cylindrical structure with octagonal symmetry, comprising coaxial rings and a central aqueous channel. Small, soluble molecules freely diffuse through the NPC but molecules over ∼40 kDa are selectively transported via active mechanisms. Active transport of protein and RNA is mediated by the karyopherin (KAP) family of nuclear transport receptors and the Ras-like GTPase Ran. There is a small family of KAP-αs, six in *Homo sapiens* and one in *Saccharomyces cerevisiae*, which recognise nuclear localisation signals (NLS) on cargo and bind to a member of the larger KAP-β family [5]. However, most transport is independent of KAP-α and mediated by direct recognition of the NLS or nuclear export signal (NES) by a KAP-β.

All functionally defined KAP-βs share a similar architecture and are extremely flexible [6], superhelical proteins composed of ∼20 consecutive HEAT (for Huntingtin, elongation factor 3, protein phosphatase 2A, and yeast PI3-kinase TOR1) repeats, each of which is composed of a pair (A and B) of antiparallel α-helices [7]. The HEAT repeats stack with a minor clockwise twist, forming an inner cargo-binding concave surface of B helices and an outer convex surface formed from the A helices (reviewed in [8]). Overall sequence similarity across the KAP-β family is low, at about 15–20%, with the N-terminal portion of the KAP-β protein, which binds the small GTPase Ran, being the most conserved region [9].

**Figure 1:**
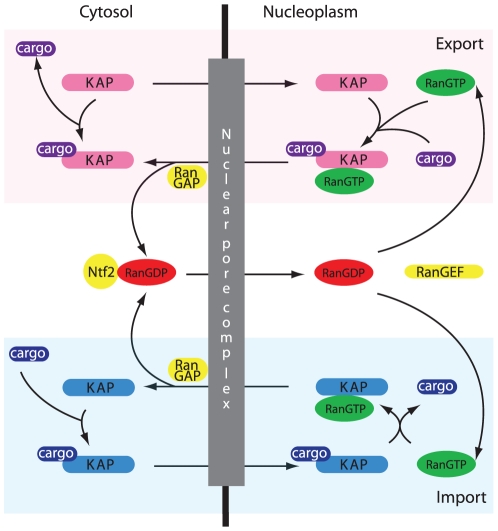
Schematic illustrating the basic functions of karyopherin-betas (KAP-βs) in context. The nuclear envelope is punctuated by nuclear pores, within which sit the proteinaceous nuclear pore complexes. Transport is bidirectional *via* a central channel and is gated by an incompletely defined mechanism. KAP-βs participate in both import (blue panel) and export (pink panel), and are also known as importins and exportins respectively. However, many KAP-βs function in both modes and hence a clear designation between import and export is not apparent. Distinct cargo are imported and exported by formation of a complex in the origin compartment; this complex dissociates on reaching the destination compartment. The RanGTP/GDP gradient, which governs directionality of transport, is maintained by the localization of RanGEF to the nucleus and RanGAP to the cytosol. RanGDP is transported to the nucleus by its own import factor, Ntf2.

Most yeast and mammalian KAP-βs are functionally classified as importins [10] or exportins [11], depending on the direction of transport they have been shown to mediate (Figure 1). Importin KAP-βs bind the cargo NLS directly or via an adaptor, e.g. KAP-α [12]. At the NPC, the KAP-β•cargo complex interacts with phenylalanine-glycine repeat-containing nucleoporins (FG-NUPs) located at the NPC central channel [13]. Upon arrival in the nucleoplasm and association with RanGTP, the KAP-β•cargo complex dissociates and KAP-β•RanGTP returns to the cytoplasm, where GTP hydrolysis dissociates the KAP-β•Ran complex. By contrast, exportin KAP-βs bind RanGTP and NES-containing cargo and the complex translocates through the NPC to the cytosol. Ran levels in the nucleus are replenished by re-import of RanGDP in complex with the nuclear import factor Ntf2 [14]. Directionality is facilitated by the Ran GTP/GDP gradient across the NE (reviewed in [15] [16]). RanGEF is restricted to the nucleus and maintains a high nuclear RanGTP concentration, while RanGAP, localised to the cytoplasmic face of the NPC or in the cytosol, depending on the organism [17], maintains a low cytoplasmic RanGTP concentration.

Several models have been proposed to explain selective translocation through the NPC, including a high density of low affinity binding sites, partitioning based on hydrophobicity or gel-like states within the channel, reduction of dimensionality by KAP binding to the FG-NUPs and more formal gating systems [18] [19] [20] [21] [22]. Recent work suggests that selectivity can arise from a balance between efficiency and speed of transport for each KAP-β•cargo complex [23]. While no consensus mechanism has emerged, FG-NUPs clearly have a major role as these disordered proteins selectively bind KAP-β complexes [24], concentrate them at the NPC and restrict passive diffusion [18]. KAP-βs themselves may also directly maintain selectivity by impeding passage of proteins that do not specifically bind FG-NUPs [25].

The KAP-β family transports an extremely broad range of molecules; tRNAs and rRNAs are carried from the nucleus to the cytoplasm while transcription factors, DNA-interacting and RNA-processing proteins are imported to the nucleus. Several pathways, such as biosynthesis of ribosomes, require components to engage in multiple crossings of the nuclear envelope [26]. While many KAP-β cargoes are known (see [27] for recent review), the full range of molecules transported by individual KAP-βs is undefined; hence KAP-βs currently classed as importins may, with additional analysis, be found to function in export. The absence of a rigorous discrimination between export or import pathways and substrate specificity may arise from a rather complex hierarchy of binding affinities. For example in *S. cerevisiae* only four KAP-βs are essential [16] and many can be deleted in combination, indicating redundancy [28]. Also, some proteins including histones [29] are imported by several different KAP-β family members, again arguing for redundancy. By contrast, Kap123p in *S. cerevisiae* is the sole KAP-β involved in import of ribosomal proteins. Confusingly, Kap123p knockouts are viable [30], but interestingly ribosomal proteins are transported by Pse1p in Kap123p knockout cells, indicating that cargo can switch from one KAP-β to another. Further, KAP-α is highly specific, associating exclusively with KAP-β1. Thus a complex relationship between specificity and flexibility of cargo recognition governs KAP-β/cargo interactions, confounding attempts to uncover evolutionary relationships based on simple genetic, functional or specificity criteria. Interestingly a similar situation of apparent redundancy, using viability in *S. cerevisiae* in rich media as the assay, is found for FG-NUPs. A considerable level of knockout is possible before loss of viability [31]. However, retention of a similar number of FG-NUPs and conserved features across eukaryotes argues that selective pressure has maintained the overall heterogeneity of FG-NUPs [32].

Structural analysis of the KAP-β member importin-β in complex with various cargo reveals that distinct molecules interact with different C-terminal sites [33] [34] [35]. Thus KAP-βs likely possess multiple binding sites for recognition and transport of the wide range of cargo. Cargo-bound states also exhibit distinct conformations, illustrating the flexibility of the KAP-β structure, which may contribute to selection and binding of the repertoire of cargo molecules. This absence of a simple relationship between sequence, structure and binding specificity, coupled to the low level of sequence conservation between KAP-βs, makes determining the evolutionary origins and history of KAP-βs challenging.

An accurate KAP-β phylogeny will reveal evolutionary relationships between functionally similar members and uncover the events leading to functional diversification. Recent data suggests deep evolutionary connections between NPC and endomembrane transport components, while broad conservation of many protein families required by the endomembrane system and the NPC suggests that much eukaryotic compartmentalisation predates the LECA [3] [36] [13] [32] [37]. In terms of nucleocytoplasmic transport, a simple KAP-β repertoire in the LECA would imply that much complexity in extant eukaryotes is lineage-specific while a conserved KAP-β repertoire across eukaryotes would suggest that nucleocytoplasmic system complexity was established in LECA.

All eukaryotes are thought to descend from one ancestor which gave rise to the six supergroups [38], known as Opisthokonta, Amoeboza, Archaeplastida, Excavata, Chromalveolata and Rhizaria. In a more recent classification, Chromalveolata and Rhizaria were proposed to be members of one supergroup ‘SAR’ (Stramenopiles+Alveolates+Rhizaria) [39]. Previous investigations of KAP evolution [40] [41] [42] [32] [43] [5] were restricted to a limited range of taxa that was biased towards animals and yeasts, members of the Opisthokonta. Specifically Mason and coworkers reconstructed evolution of the KAP-α family, determining the presence of an ancient KAP-α1/KAP-α1-like subfamily with evidence for lineage-specific expansion into KAP-α2 and KAP-α3 forms in the Opisthokonta and further expansions and secondary losses in Metazoa [5]. These authors suggested that a system utilizing KAPα was likely the ancestral configuration, with KAP-α-independent pathways arising later. However, the analysis could not predict events prior to establishment of the Opisthokonta. In a broader study, Mans *et al* [41] suggested that while there were ∼13 KAP-β subfamilies, only six or seven of these were identified within the alveolates and trypanosomatids, suggesting that much KAP-β evolution was lineage specific. We considered that re-evaluation of the KAP-β repertoire using a broader range of genomes together with iterative searches would result in more extensive KAP-β sampling with improved understanding of their origins and subsequent evolutionary history. Our findings are consistent with much KAP-β complexity being established by the time of the LECA. Significant expansion, lineage-specific innovation and secondary losses are also in evidence.

## Results and Discussion

### Karyopherin-β is represented by at least fifteen subfamilies

To examine sequence relationships within the KAP-β family across the eukaryotes, we performed a semi-automated search of a panel of predicted proteomes. Species selection was designed to cover the full range of eukaryotic diversity possible with current genomic sampling, thus revealing lineage-specific patterns of gene conservation and identifying lineage-specific expansions and losses.

**Figure 2:**
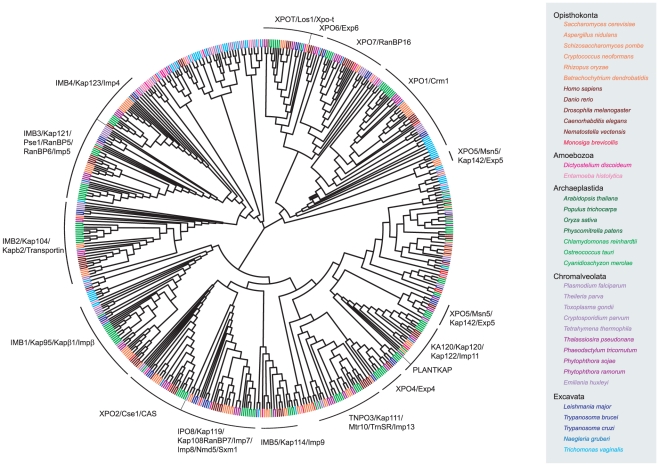
Neighbour-joining tree of KAP-β sequences across eukaryotes. Six hundred and twenty two KAP-β candidate sequences, retrieved from 36 completed predicted proteomes, and representing five of six established eukaryotic supergroups, were clustered into a NJ tree with ClustalW. Taxa are coloured by species, listed on right, and by eukaryotic supergroup. All sequences highlighted by a black arc at the rim of the tree exhibit evidence for specific KAP-β subfamily membership and are located on a branch immediately adjacent to at least one other similar taxon on the tree. Unhighlighted sequences either have some evidence for sub-family membership but are not clustered, or are orphans. The subfamily name of each cluster is followed by additional names, based upon *S. cerevisiae* and *H. sapiens* gene names [16]. Tree drawn using PhyloWidget [89].

Three rounds of reciprocal BLAST [44] scans were performed using known KAP-β query sequences [9] [15] [16]. All hits from the first BLAST scan with an e-value less than 10^−10^ were also collected. PSI-BLAST [45] scans were performed using pfam [46] domains IBN_N and Xpo1, which are specific for several KAP-β family members. All returned sequences were then pooled and sequences showing no evidence of KAP-β family membership were removed from the dataset. Following these searches, 630 sequences meeting criteria for KAP-β membership (see methods) were retrieved and 622 of these sequences were subjected to the analysis presented in this section. For reasons of computational tractability, bootstrapped neighbour-joining (NJ) analysis was used to produce an initial subfamily classification system in which any sequences with similar BLAST results and located on adjacent branches of the NJ tree were counted as a cluster. All the subfamily assignments made in this analysis were, where possible, confirmed by formal phylogenetic methodology (see following sections). This preliminary clustering is shown in Figure 2 and together with statistical support in File S1. Fifteen subfamilies, each containing representatives from three or more eukaryotic supergroups, were identified. Each cluster was named using the UniProt ID of either an *S. cerevisiae* or *H. sapiens* KAP-β as follows: IMB1, IMB2, IMB3, IMB4, IMB5, XPO1, XPO2, XPO4, XPO5, XPO6, XPO7, XPOT, IPO8, KA120 and TNPO3. All subfamilies were represented by a single cluster except XPO5, represented by two clusters, which may arise from high sequence diversity. Additional NJ clustering with a sequence subset (composed of the four reference sequence sets, see methods) of each KAP-β subfamily plus all XPO5 candidates produced a single XPO5 cluster, indicating that XPO5 candidate sequences likely comprise a single subfamily (data not shown). Significantly, a well supported cluster of Embryophyte-specific (land plant) sequences was identified (File S1) and designated PLANTKAP.

Following the identification of 15 subfamilies, each sequence in the dataset was assigned candidate membership to either a KAP-β subfamily or PLANTKAP, or as an orphan, *i.e.* unassignable to a subfamily. *S. cerevisiae* Pdr6 (Kap122) was the sole functionally validated KAP-β failing to map to a KAP-β subfamily. Further BLAST analysis revealed *H. sapiens* IPO13 and additional divergent TNPO3 subfamily members as Pdr6 closest relatives. NJ analysis with selected KAP-β subfamily representatives (see methods), all TNPO3 candidates plus Pdr6 and its orthologues, resulted in Pdr6 clustering with the majority of TNPO3 candidates (data not shown), and therefore Pdr6 was classified as a candidate for belonging to the TNPO3 subfamily.

For 30 of the 36 genomes searched, all KAP-β candidates were assignable. In the remaining genomes, the orphan KAP-β sequences were all detected using PSI-BLAST-based domain-specific scans. These sequences may correspond to recent taxon-specific innovations or represent highly diverged representatives of established KAP-β subfamilies. They were not studied further. It is possible that not all KAP-βs were captured by our search; Pdr6 would not have been included if not an initial query. Therefore, whilst exhaustive, we cannot exclude the possibility that additional KAP-β sequences were not identified, and KAP-β complexity may exceed that sampled here. However, the search did correctly identify all KAP-βs detected as NE-associated by proteomics in *Trypanosoma brucei* [32], suggesting that the dataset is very comprehensive, and only likely to have missed extremely divergent candidates.

In summary, we identified at least 15 KAP-β subfamilies containingone or more sequences from at least three eukaryotic supergroups. In the absence of a convincing root of the eukaryotic tree [47], this distribution is best interpreted as representing an ancient presence in eukaryotes. The most likely interpretation therefore is that these KAP-β subfamilies were established before the eukaryote radiation.

### Karyopherin-β family evolution prior to eukaryotic expansion

**Figure 3:**
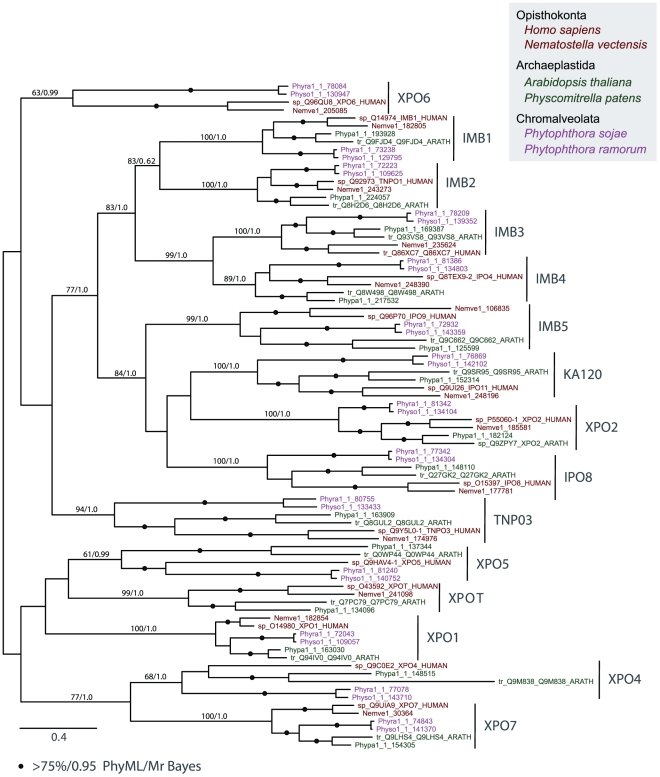
Phylogeny of selected representatives of the fifteen KAP-β subfamilies. Numbers on internodes refer to PhyML bootstrap support/MrBayes posterior probability values and the MrBayes topology is shown. Dots indicate values better than 75% bootstrap support and 0.95 posterior probability, while full values are given for important internodes supporting KAP subfamilies. The colour scheme is as in figure 2 and species included are as follows: *Homo sapiens* (HUMAN), *Nematostella vectensis* (Nemve), *Phytophthora ramorum* (Phyra), *Phytophthora sojae* (Physo), *Arabidopsis thaliana* (ARATH) and *Physcomitrella patens* (Phypa). Subfamilies are indicated by vertical bars and inidvidual sequences are represented by gene IDs.

For phylogenetic analysis, a reduced set of KAP-β subfamily representatives were selected (Figure 3). We retained sequences from four supergroups where possible to ensure broad representation and hence validate the subfamily presence within the LECA, and included the following taxa: Opisthokonta: *Homo sapiens, Nematostella vectensis;* Archaeplastida: *Arabidopsis thaliana, Physcomitrella patens*; Chromalveolata: *Phytophthora ramorum, Phytophthora sojae*; Excavata: *Leishmania major, Trypanosoma brucei.* Representatives of the Amoebozoa were excluded as several sequences from this supergroup were found to be more diverged in an initial analysis (data not shown). Where statistical support was poor, the more divergent Excavata sequences were removed.

The presence of the N-terminal IBN_N pfam domain (e-value threshold <0.1) in members of every subfamily argues for KAP-β being monophyletic (see File S1). We sought further support by testing if each subfamily can detect all other subfamilies based on sequence homology and scanned the human proteome with PSI-BLAST aligments for each subfamily, constructed from the taxa selected above. While each subfamily was not found to detect all other subfamilies, scans with both the XPO1 and XPO5 alignments detected members of all 15 subfamilies as top hits (data not shown), supporting the hypothesis of a monophyletic origin for KAP-β.

**Figure 4:**
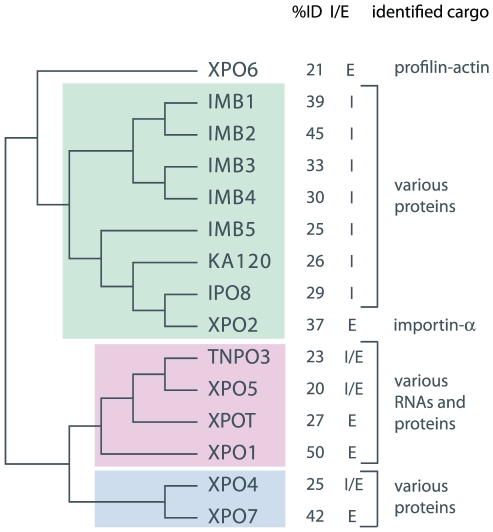
Unrooted karyopherin-β subfamily phylogeny. Schematic illustrating inferred ancestral relationships between the KAP-β subfamilies, percent identity (%id) values, known roles as import or export factor (I/E) within each subfamily and description of cargo types. This unrooted topology was inferred from a series of phylogenetic reconstructions available in Figure S1. Colored panels highlight three clades of related subfamilies whose phylogenies were initially determined; a subfamily representative of each of these clades, and of XPO6, were then used to infer a family-wide phylogeny.

An initial analysis containing representatives from all 15 KAP-β subfamilies (Figure 3, Figure S1(a)) identified 2 robust clades, supported by maximum likelihood (bs >70%) and Bayesian (pp >0.95) algorithms. XPO4 and XPO7 share a common ancestor (Figure 4 blue), as do the two clades of IMB1, IMB2, IMB3 and IMB4 and IMB5, KA120, IPO8 and XPO2. Further analysis (Figure S1(b)) resolved the relationships within this latter grouping (Figure 4 green). Two subsequent analyses of the remaining sub-families (Figures S1(c), (d)) established additional larger KAP-β family clades (Figure 4 pink). Finally, A fifth phylogenetic analysis (Figure S1(e)) established the phylogenetic relationships of four ancestral subfamilies comprising the three groups identified above (Figure 4) and XPO6. This phylogeny demonstrates that (i) XPO6 and the IMB1, IMB2, IMB3, IMB4, IMB5, KA120, IPO8, XPO2 group are descended from a common ancestor and (ii) the XPO4, XPO7 group and the TNPO3, XPO5, XPOT, XPO1 group are also descended from a common ancestor. These two groups in turn are predicted to be descended from an ancestral KAP-β. While this analysis has established a phylogeny of KAP-β subfamilies, it is not possible to determine the order in which the subfamilies diverged from their common ancestor due to the absence of a prokaryotic homologue with which to root the tree. Significantly, there is some correspondence between the phylogenetic groupings described above and published functional characteristics of KAP-β subfamily members. However, given that a complete characterisation of KAP-β function in any organism has yet to be reported, this remains tentative.

With the exception of exportin XPO2, the IMB1 clade contains KAP-β subfamilies, characterised as exclusively involved in protein, and not RNA, nuclear import. Cargoes for this subfamily include mRNA binding proteins, ribosomal proteins, histones and signal recognition particle proteins. IMB1 imports cargo associated with importin-α and XPO2 exports importin-α after cargo has been released in the nucleus.

The TNPO3, XPO5, XPOT, XPO1 clade members are functionally more diverse, based on present data. Cargoes include tRNAs, small noncoding RNAs, ribosomal subunits and proteins. XPOT and XPO1 function in export of tRNAs and proteins containing leucine-rich nuclear export signals respectively. TNPO3-like and XPO5 proteins participate in both import and export [48] [49]. As these KAP-β subfamilies are located on adjacent leaves in our phylogeny, we speculate that the ancestor of TNPO3 and XPO5 possessed a dual import/export role. The *S. cerevisiae* XPO5, Msn5p, mediates import and export of distinct cargoes [48], importing replication protein A and exporting a variety of phosphoproteins. Metazoan XPO5 representatives are responsible for export of eukaryotic elongation factor 1A (eEF1A), tRNAs [50], 60S ribosomal subunits [51] and short miRNA precursors [52] [53]. While the *S. cerevisiae* and *H. sapiens* orthologues bind dsRNA templates, functional divergence has been demonstrated by measurement of cargo binding affinities [54]. The human TNPO3-related protein IPO13 similarly imports and exports different cargoes; RBM8, Ubc9 and Pax6 are imported, and translation initiation factor eIF1A is exported [49] [55]. However, TNPO3 orthologues from *S. cerevisiae* and *H. sapiens* are only documented so far as being involved with import, carrying mRNA-binding splicing factor SR (serine/arginine-rich) proteins into the nucleus [56] [57].

The two members of the remaining clade, XPO4 and XPO7, are functionally distinct. XPO4 exports eIF-5A (eukaryotic translation initiation factor 5A) [58] and transcriptional modulator Smad3 [59] and also imports a different cargo, Sox transcription factors [60]. XPO7 exports proteins with broad substrate specificity using nuclear export signals that, unlike leucine-rich XPO1 signals, include folded motifs [61]. The remaining KAP-β subfamily, XPO6, exports profilin-actin complexes [62]. Given that our understanding of KAP-β function is incomplete, any conclusions based on correspondences between functional and phylogenetic groupings remain speculative.

To attempt to gauge levels of sequence divergence within each subfamily, percent identity values were calculated for subfamily-specific alignments of the sequences used in the phylogenetic analysis above (Figure 4). XPO5 appears to be the least constrained, which correlates with the observed functional divergence. XPO1, with a percent identity value of 50, appears to be the most evolutionarily constrained.

As we consider convergent evolution unlikely, we propose that the entire KAP-β family descended from an ancestral form. As the phylogeny is unrooted, the position of this ancestral KAP-β remains unknown, and therefore the order of events involved in elaboration of this gene family is unclear. The difference in PID values for each subfamily indicates that selective pressures are variable across the family and that any assumptions about the position of a common ancestor cannot be inferred from branch length. The common ancestor most likely functioned in both import and export, as well as transporting a broad range of cargo. As the XPO1-containing clade (Figure 4 green) both imports and exports a broad range of cargo, we suggest that the root of the tree may lie within this clade. XPO1 and XPO5 robustly detect all other subfamilies by PSI-BLAST, which suggests that these two subfamilies are the most canonical, and other subfamilies may have diverged more from the ancestral KAP-β. In an alternative model [41] [5], it was argued that KAP-α-mediated transport was the ancestral mode, and that later KAP-α-independent pathways are a later simplification. This model implies that the root lies between IMB1 and remaining members of the KAP-β family. While we cannot exclude it, we do not favor this model as it suggests that IMB1 has undergone no expansion whatsoever, while the remaining KAP-β family exhibits huge diversification. However, resolution between the two models is not possible from the data presently available. Regardless of which model is correct, clearly diverse KAP-β pathways were present in the LECA.

With a larger selection of genomes, this analysis confirms, clarifies and expands upon the evolutionary relationships for KAP-βs described previously [41] [32]. The new phylogeny (Figure 4) suggests a likely evolutionary path for the development of the KAP-β transport receptor in the transitional period between the first and last eukaryotic common ancestors.

### Karyopherin-β representation across the eukaryotic supergroups

**Figure 5:**
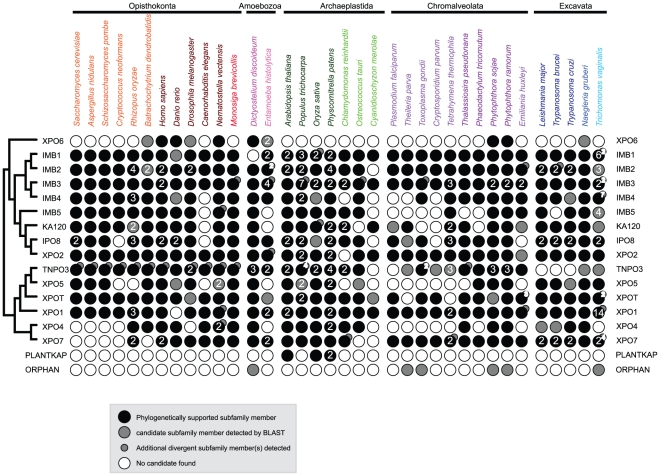
Subfamily distribution of karyopherin-βs across the Eukaryota. Black circles indicate presence of a phylogenetically supported (see methods) KAP-β subfamily member. Grey circles indicate candidate subfamily members that could not be verified phylogenetically. Empty circles indicate no candidate found. Numbered circles indicate cases where more than one candidate is found. A small circle indicates candidate(s) in addition to phylogenetically supported candidate(s) indicated by big circles. The left panel illustrates the phylogenetic relationships between subfamilies. See Table S1 for additional information including protein identifiers.

**Figure 6:**
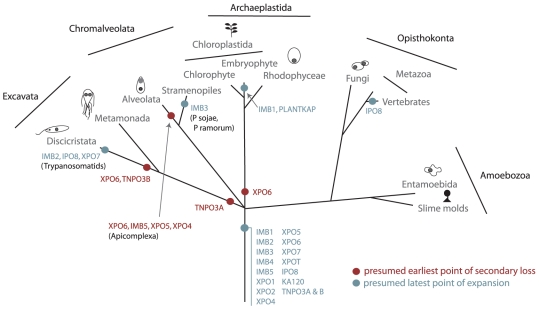
Schematic illustrating lineage-specific events in KAP-β family evolution. Proposed positions of origin and secondary loss are shown on a schematic eukaryotic phylogeny, representing five major supergroups. Dots indicate expansions and losses; note the position in an internode is arbitrary, and only events that are shared by more than one taxon are shown. TNPO3 is proposed to have undergone an ancestral duplication (A and B) followed by loss in the lineage leading to the modern Excavata. Note that the more recently accepted SAR supergroup, encompasing stramenopiles, alveolates and Rhizaria is used here. Figure adapted from [90].

Our initial search produced over 600 KAP-β family members of which, for computational reasons, only a subset were included in the pan-eukaryotic phylogenetic reconstruction (Figure 4). We confirmed subfamily membership for the remaining KAP-βs by additional analysis using Bayesian methods (see methods, Table S1, Figure 5). Sequences shorter than 50% the length of validated KAP-β proteins were excluded. The analysis confirmed representation of all KAP-β subfamilies in all supergroups, with the established exception of XPO6 in Archaeplastida. While some species possess one or more phylogenetically verified members of each KAP-β subfamily, others have divergent representatives, and for some species entire subfamilies are absent (Figure 6).

A striking feature is the frequency of secondary loss in individual species, suggesting that many organisms sculpt nuclear transport by elimination of KAP-β subfamilies. While we cannot exclude failure to detect highly divergent KAP-β sequences as an explanation, we consider that our searches sufficiently exhaustive to preclude this as a general explanation and that most losses are genuine. With the continuing and increasing availability of completed genomes, this analysis may be improved by including more species, particularly we note the completion of the Chromalveolata Stramenopile *Ectocarpus siliculosus* [63] and *Aureococcus anophagefferens* [64] genomes which were not available at the time of beginning this study.

Amongst supergroup-restricted losses, the most prominent is absence of XPO6 from Archaeplastida; as seven Archaeplastida species were included this is unlikely a sampling or data issue. XPO6 exports actin [62], but XPO1 can also perform this function [65], and assumes this role in plants. Significantly, XPO6 is also lost from many other lineages, suggesting that its function is dispensable under certain contexts.

Within supergroups, some taxon groupings exhibit notable KAP-β divergence. In Opisthokonta, several fungi have lost XPO4 and XPO7. Multicellular organisms have, in general, maintained the full complement of KAP-β subfamilies, while unicellular organisms are more likely to have undergone loss or great sequence divergence. A clear exception is the minimized KAP-β system found in nematodes, as *Caenorhabditis elegans* appears to have ∼50% of the KAP-βs from the IMB1 clade. This result was confirmed for *C. briggsiae* (data not shown), and indicates that a full KAP-β complement is not necessary for multicellularity.

In Archaeplastida, higher plants have undergone several subfamily expansions, with no evidence of secondary loss apart from that of XPO6. By contrast, amongst unicellular Archaeplastida, secondary losses are common, with nine of fifteen subfamilies lost from the hot-spring red alga *Cyanidioschyzon merolae*. This organism has a very small gene complement [66] and is an extremophile, therefore the result is not unexpected.

In Chromalveolata, the Apicomplexa (*Cryptosporidium parvum, Toxoplasma gondii, Theileria parva, Plasmodium falciparum*) have undergone similar patterns of secondary loss (XPO6, IMB5, XPO5, XPO4), suggesting that these were lost in their common ancestor. Multiple losses in endomembrane transport are reported for Apicomplexans, suggesting a significant degree of divergence in transport pathways in general in these taxa [67] [68] [69].

Within Excavata, only XPO6 and TNPO3 are lost from the kinetoplastids, consistent with retention of other trafficking systems by this supergroup [67] [68]. Significantly, *Trichomonas vaginalis* has expanded multiple KAP-β families, a feature of interest as specific expansions of multiple gene families involved in intracellular trafficking, including Rabs [70] [71] and adaptins [72] [71], have also been described. This suggests that KAP-β may be a component of the expanded gene cohort in this organism. It is unclear why such expansions occurred [70].

### Lineage-specific expansions

Many examples of species-specific duplications or expansions were found, the most dramatic being fifteen XPO1 subfamily members in *T. vaginalis*. In addition, several duplications (Figure S2 and Figure 6) are predicted within individual supergroups as follows:

- A common ancestor to *H. sapiens* and *D. rerio* duplicated IPO8 (Figure S2(a)).
- A common ancestor to land plants duplicated IMB1 before full diversification (Figure S2(b)). While both IMB1 subclades contain higher plants, one contains a duplicated *P. patens* (moss) sequence while the second branch lacks a moss representative, presumably from secondary loss.
- There are two versions of IMB3 in *P.* sojae and *P. ramorum* (Figure S2(c)), suggesting a duplication in a Chromalveolata common ancestor. However it was not possible to produce a robust topology phylogenetically and so any conclusions are tentative.
- One or more common ancestors to the Kinetoplastida duplicated each of XPO7, IPO8 and also may have duplicated IMB2 (Figure S2(d, e, f)). For XPO7, both paralogues are divergent while for IMB2, just one paralogue is diverged. The *T. brucei* paralogue of the more diverged IMB2 clade (Tb10.6k15.3020) was identified as a component of the NPC proteome [32], providing direct evidence that this KAP-β is functional. While this paralogue may have arisen by duplication in a common ancestor, this is not confirmed by phylogenetic analysis and so any conclusions are tentative.
- The novel clade, PLANTKAP, is restricted to land plants. Both bootstrapped NJ and Bayesian algorithms placed PLANTKAP close to IPO8 (data not shown and Figure S2(g), suggesting these have diverged from the IPO8 subfamily. *Populus trichocarpa* contains only a truncated PLANTKAP (accession Poptr1_1_724002), likely a sequencing artifact. Several land plants have two IPO8 KAP-β paralogues, but these are derived from recent species-specific duplications (data not shown).
- Except for Excavata, all supergroups possess a duplicated TNPO3. The simplest explanation is an ancestral duplication and Excavata secondary loss. When all TNPO3 candidate sequences were clustered by NJ, a cluster of robust candidates identified by phylogenetic analysis (data not shown), was formed with more diverged candidates being excluded (Figure S2(h)). This divergent TNPO3 group includes *S. cerevisiae* Pdr6 and *H. sapiens* IPO13. While IPO13 is involved in both import and export [49], Pdr6 is only noted as involved with import [73]. The more diverged Archaeplastida TNPO3 group only contains land plant representatives and remains sufficiently closely related to TNPO3 that these sequences validate as TNPO3 subfamily members by phylogeny. Therefore, if TNPO3 is comprised of two subfamilies, this lesser degree of divergence in plants and higher degree of divergence in Opisthokonta potentially reflects differing selective forces between the eukaryotic lineages.
- A single example of KAP-β innovation by gene fusion was identified. *P. sojae* and *P. ramorum* contain an XPOT::ABC-type transporter chimera, which likely arose in their common ancestor. It remains unknown if these proteins actively function as KAP-βs or as proteins with novel function.

Overall, there is clear evidence for specific, but limited, secondary losses and lineage-specific expansions within the evolutionary history of the KAP-β family. There is however little evidence for major evolutionary innovation within the KAP-β family post-dating the LECA.

### Conclusions

Employing a combination of domain searching and iterative BLAST analysis, we identified over six hundred KAP-β genes from a broad range of eukaryotes. Due to a shared IBN_N pfam domain and the fact that all subfamilies are returned as top hits in XPO1 and XPO5 PSI-BLAST searches, we conclude that the KAP-β family most likely arose by divergent evolution, *i.e.* from a single ancestral KAP-β. Cluster analysis identified fifteen KAP-β subfamilies that, except XPO6 in Archaeplastida, are represented in all eukaryotic supergroups, and hence were likely present in the LECA. This also suggests that KAP-β transport mechanisms have been conserved since the LECA, consistent with conserved NPC composition and additional aspects of the nuclear envelope [32] [4]. Further, a derived evolutionary history successfully places the vast majority of KAP-β subfamilies into three major clades, for which there is some functional support.

The IMB1 clade is responsible for protein, and not RNA, transport. Seven of the eight subfamilies in the clade are importins, together with KAP-βs responsible for KAP-α import and export (IMB1 and XPO2). The XPO1 clade is involved in both import and export of both RNA and proteins. While these phylogenetic groupings may reflect deep functional relationships, this remains to be confirmed by further experimental work. Several lineage-specific events were identified, most notably PLANTKAP, a cluster of plant-specific KAP-βs likely derived from IPO8, and a TNPO3 subfamily expansion which may indicate an additional KAP-β subfamily. We also found several examples of secondary loss, many of which clearly occurred early in evolution of specific supergroups while some are more recent. However, most significantly, there is little evidence for large paralogous expansions within either individual taxa or supergroups, suggesting that the overall configuration of the KAP-β family has been retained during post-LECA evolution.

## Methods

### Identification of candidate karyopherin-β sequences

A panel of thirty six predicted proteomes representing as wide a range of eukaryotic diversity as possible (all supergroups except Rhizaria, for which there is no available no genome sequence) and restricted to completed genomes, was assembled from the following species: *Arabidopsis thaliana, Aspergillus nidulans, Batrachochytrium dendrobatidis, Caenorhabditis elegans, Chlamydomonas reinhardtii, Cryptococcus neoformans, Cryptosporidium parvum, Cyanidioschyzon merolae, Danio rerio, Dictyostelium discoideum, Drosophila melanogaster, Emiliania huxleyi, Entamoeba histolytica, Homo sapiens, Leishmania major, Monosiga brevicollis, Naegleria gruberi, Nematostella vectensis, Oryza sativa, Ostreococcus tauri, Plasmodium falciparum, Phaeodactylum tricornutum, Phytophthora ramorum, Phytophthora sojae, Populus trichocarpa, Physcomitrella patens, Rhizopus oryzae, Saccharomyces cerevisiae, Schizosaccharomyces pombe, Trypanosoma brucei, Trypanosoma cruzi, Tetrahymena thermophila, Thalassiosira pseudonana, Theileria parva, Toxoplasma gondii* and *Trichomonas vaginalis.* See Table S1 for sources of raw data. The panel was searched for candidate KAP-β sequences with BLAST [44] and KAP-β-specific domains with PSI-BLAST [45].

For the BLAST scans, functionally validated KAP-β sequences [9] [15] [16] and their *S.* cerevisiae or *H.* sapiens orthologues (identified as reciprocal best BLAST hits) were used as queries in BLASTp scans of the predicted proteome panel. All reciprocal best BLAST hits (i.e. the hit, when used as query, returned the original query as top hit or with identical e-value as the top hit), were collected and used as query sequences in a second BLASTp scan of the panel. All reciprocal best BLAST hits were collected and used as query sequences in a third BLASTp scan. All returned reciprocal best BLAST hits, together with all hits in the initial BLAST scan with e-value <10^−10^, were collected. An additional eight hits from the initial BLAST scan with an e-value of less than 0.01, were selected on the basis of (i) being greater than 500 amino acids in length and (ii) fold recognition by FUGUE where sequences were counted as candidates if a KAP-β family member was returned as top hit with ZSCORE greater than 6.

For the PSI-BLAST scans, Pfam [46] domains IBN_N (PF03810, Importin-β N-terminal) and Xpo1 (PF08389, Exportin 1-like protein), which are specific to several KAP-β family members, were used as query sequences in blastpgp (PSI-BLAST) [45]) searches. This was carried out as follows: Multiple sequence alignments (MSAs) for each domain were retrieved from the pfam website [74] and realigned with Muscle [75]. Sequences with greater than 90% identity were removed using Jalview 2.06 [76]. For each member sequence of a pfam-domain query (reduced-redundancy alignment), PSI-BLAST was to scan the predicted proteome panel. The input of each PSI-BLAST scan included the pfam domain alignment (*ie*, ‘jump-start’ from MSA mode) and the maximum number of rounds was set to three. For sequences retrieved with e-value of 0.0001 or less in any of the PSI-BLAST scans, the sequence segment giving the lowest e-value was identified. These segments were scored as valid matches if they, or the whole predicted coding sequence from which they were derived, satisfied any of the following criteria: (i) whole sequence annotated in UniProt [77] as containing the query domain according to pfam or InterPro [78], (ii) sequence segment detected a protein containing the domain, according to pfam or InterPro, as the highest scoring retrieved sequence when used as BLAST query against S. cerevisiae, or (iii) whole sequence gives an e-value <1.0 for the domain when used as hmmpfam [79] query against the pfam version 18.0 HMM database. The majority (>90%) of retrieved sequences for both IBN_N and Xpo1 domains passed the validation test. Having verified that the PSI-BLAST-based domain detection strategy did not detect a significant number of off-target sequences, the full sequences from all hits, regardless of validation status, were collected.

Redundant sequences were identified by all-vs-all BLAST and discarded. Criteria for removal of off-target sequences from the pooled dataset were set after a preliminary analysis by visual inspection of a ClustalW [80] neighbour-joining (NJ) tree of all sequences in which the treefile labels had been annotated. Annotations included BLAST and PSI-BLAST results, predicted protein size, predicted charge (using pepstats of the EMBOSS package [81]), predicted domain content and, in cases with no evidence of IBN_N or Xpo1 domains by hmmpfam prediction with no threshold, detection of KAP-β homology by fold recognition. The eight low-scoring BLAST hits were removed from the NJ analysis as they introduced distortions in the NJ tree.

Sequences meeting either of the following criteria were removed:

- unexpected hmmpfam-predicted domain content and no evidence of hmmpfam-predicted IBN_N or Xpo1 domains (e-value <0.1), or
- no evidence of IBN_N or Xpo1 domains by hmmpfam prediction with no threshold and sequence matched no KAP-β family members by fold recognition with FUGUE's fugueseq [82] (ZSCORE cutoff of 0).

For reciprocal BLAST round three matches, sequences matching only the first condition of (ii) were also removed. In one exception to (ii), a sequence without FUGUE or hmmpfam evidence for family membership (XPO5 candidate Poptr1_1_241008) was retained as it clustered with other XPO5 candidates in the ClustalW NJ tree that was part of the preliminary analysis. Three HEAT domain-containing sequences that did not match either of the criteria were also discarded: Nemve1_128552, as it was detected only by a query sequence that was itself rejected, and sp_Q7Z460-1_CLAP1_HUMAN and tr_A8WHM7_A8WHM7_DANRE, which are annotated as belonging to a different gene family. An additional two sequences (tr_Q54TU2_Q54TU2_DICDI and ent_h_54.m00221) were also removed; while both matched KAP-β family members by fold recognition with FUGUE's fugueseq, the KAP-βs were not the top match and the sequences are probably members of a different gene family. Also, both were much longer than expected for KAP-βs.

### NJ cluster analysis of Karyopherin-β candidate sequences

The remaining 622 KAP-β candidate sequence were clustered in a ClustalW [80] NJ tree. The eight low-scoring BLAST hits, which were included in further analyses, were excluded from this NJ analysis as they were only weakly detected by BLAST and introduced distortions in the NJ clustering tree. A small number of sequences were trimmed before this step as they were substantially larger than the other candidates, containing additional sequence with no KAP-β homology – this is assumed to be the result of sequence assembly/annotation issues in the original databases. The treefile labels were then annotated with the same data used for the preliminary analysis. Where possible, each taxon was assigned, by hand, to a subfamily on the basis of subfamily membership of the BLAST query which first detected the sequence during the initial BLAST scan and three rounds of reciprocal BLAST. Any taxon located on a branch immediately adjacent to at least one other taxon with the same subfamily assignment was classed as being a member of that cluster. Taxa with no indication of KAP-β subfamily membership were assigned as ‘ORPHAN’.

### Phylogenetic analysis of the karyopherin-β family

Multiple sequence alignments were generated using Muscle and edited in Jalview 2.4 as follows: Alignments were coloured by conservation with no threshold (this is the default ‘colour by annotation’ setting) and then uncoloured columns and less-conserved columns (conservation value of 1) that were at the junctions of well-conserved blocks of sequence were removed. Phylogenetic analysis was performed using MrBayes [83] and PhyML [84]. All calculations were performed on CamGrid [85]. MrBayes run parameters were prset aamodelpr = mixed; lset rates = gamma Ngammacat = 4; mcmc ngen = 1000000; samplefreq = 1000; nchains = 4; startingtree = random; sumt burnin = 100. PhyML parameters were nb bootstrapped data sets = 100; substitution model =  WAG; proportion invariable sites = 0.0; nb categories = 4. For each PhyML analysis, ProtTest [86] was used to determine the appropriate substitution model and gamma parameter.

### Phylogenetic verification of subfamily membership

Datasets for verification of subfamily membership using phylogenetic analysis were generated by adding the sequences of interest to the appropriate one of four reference sequence sets which were each composed of sequences from selected species and from one of four KAP-β subfamily groupings only. The reference sequences were previously used for establishment of KAP-β subfamily phylogeny and were from the following supergroups: Opisthokonta (*H. sapiens, N. vectensis*), Excavata (*L. major, T. brucei*), Chromalveolata (*P. ramorum, P. sojae*) and Archaeplastida (*A. thaliana, P. patens*). The four reference sequence files contained the following KAP-β subfamilies: IMB1/IMB2/IMB3/IMB4, IMB5/KA120/IPO8/XPO2, TNPO3/XPO5/XPOT/XPO1 and XPO4/XPO7/XPO6. Trees were generated using MrBayes with the same parameters as described above. Sequences were counted as phylogenetically verified subfamily members if they were located in the expected branch of the tree with statistical support (posterior probability >95%), regardless of branch length or position on the branch.

### Calculation of percent identity for each KAP-β subfamily

A multiple sequence alignment for each KAP-β subfamily was generated in Muscle using only selected sequences from the species used for the reference dataset. Each MSA was used to generate two subalignments; the first contained sequence from *H. sapiens*, *P. sojae* and *A. thaliana* and the second contained sequence from *N. vectensis*, *P ramorum*. and *P. patens*. In some cases the alignment contained only two sequences as the third sequence was absent or was considerably diverged from canonical KAP-β sequence. Percent identity for each subalignment was calculated by alistat from the HMMer package [79]. The results for the two subalignments for each KAP-β subfamily were averaged after confirming that the results for each subalignment were similar (within 2% of each other).

### Automation

Sequence retrieval, BLAST scans, identification, validation and annotation of hits and all associated parsing of text files were carried out in batch mode with scripts written in Perl5 [87], using modules from the BioPerl library [88] and are available from the authors on request.

## Supporting Information

Figure S1 Phylogenetic trees used for establishment of KAP-β phylogeny.
(PDF)

Figure S2 Phylogenetic trees constructed using MrBayes showing lineage-specific expansions and NJ tree containing all TNPO3 candidate sequences, see main text for details.
(PDF)

Table S1 **Details of data sources and data from analysis of karyopherin-β families.** (a) Sources for predicted proteome data. (b) Table listing IDs for candidates for each species and KAP-β subfamily. This dataset was used to generate Figure 4. Includes annotation indicating if the candidate clustered in the NJ tree of Figure 2, if the candidate was a reciprocal BLAST best hit for a known KAP-β and if the candidate was assignable to a sub-family phylogenetically.
(XLS)

File S1 Treefile used to generate Figure 2 - ClustalW neighbour-joining tree of 622 known & candidate karyopherin-betas. Suggested tree viewing software: http://www.phylosoft.org/archaeopteryx/. Each taxon is annotated as follows: gene_name or UniProt ID; *length; *pfam domain predictions (e-value <0.1); *BLAST results (_q indicates that the sequence was used as a query in the first BLAST round, _b1, _b2, _b3 indicate that the sequence was picked up in best reciprocal BLAST hit round 1, 2 or 3, _b0 indicates that the sequence was picked up in the first round of BLASTs with an evalue <10e-10); * hmmpf (if IBN_N or Xpo1 was detected in the sequence by hmmpfam with no e-value threshold); * PB (if the sequence was detected in either of the PSI-BLAST IBN_N or Xpo1 domain scans; ch 00.00 charge calculated by pepstats; FUGUE 00.00 (If the sequence matched a karyopherin-beta family member according to fugueseq and ZSCORE of top match). Note - FUGUE results for selected sequences only; *subfamily assignment. Subfamily assignments:__NAME_1, for candidate NAME subfamily candidates clustered with other candidate NAME subfamily candidates; __NAME_0 for candidate NAME subfamily candidates not clustered with other candidate NAME subfamily candidates; __ORPHAN for karyopherin-beta candidates with no subfamily assignment.
(TXT)
