# Supplementary material for: Evolution of the Karyopherin-β Family of Nucleocytoplasmic Transport Factors; Ancient Origins and Continued Specialization
Source: PLoS One. 2011 Apr 27;6(4):e19308. doi: 10.1371/journal.pone.0019308 (PMC3083441; doi:10.1371/journal.pone.0019308)
Supplement: Figure S1 — Phylogenetic trees used for establishment of KAP-β phylogeny. (PDF) [file pone.0019308.s001.pdf]

Figure S1

Phylogenetic analysis for establishment of phylogeny of Karyopherin-beta family members.
Numbers on trees are posterior probability (MrBayes) and bootstrap support (PhyML) values.
Phylogenetic relationships were inferred only when supported by MrBayes (posterior probability >95%) and PhyML trees (bootstrap value >70%).
A schematic of the inferred phylogeny is shown on this page and schematics of inferred relationships are shown for each pair of trees in (a-e) below.

**Species ID**	**Species**
Hs	*Homo sapiens*
Nv	*Nematostella vectensis*
At	*Arabidopsis thaliana* (cultivar Columbia)
Pp	*Physcomitrella patens* ssp patens ecotype Gransden 2004
Ps	*Phytophthora sojae*
Pr	*Phytophthora ramorum*
Lm	*Leishmania major*
Tb	*Trypanosoma brucei*

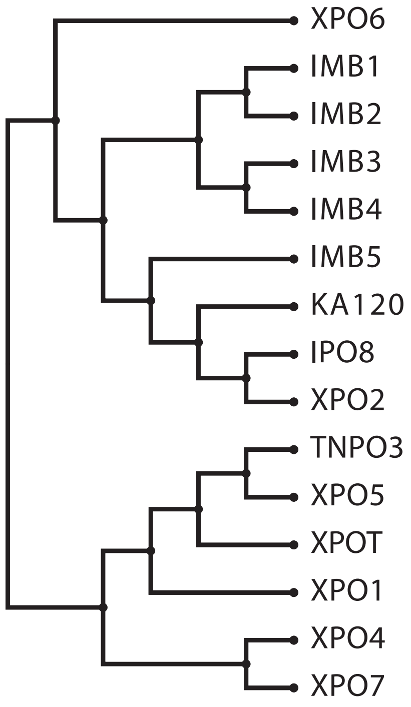

short ID	long ID	short ID	long ID
Hs_XPO6	sp_Q96QU8_XPO6_HUMAN	Pp_IPO8	Phypa1_1_148110
Nv_XPO6	Nemve1_205085	Ps_IPO8	Physo1_1_134304
Ps_XPO6	Physo1_1_130947	Pr_IPO8	Phyra1_1_77342
Pr_XPO6	Phyra1_1_78084	Lm_IPO8	LmjF13.0640
Hs_IMB1	sp_Q14974_IMB1_HUMAN	Tb_IPO8	Tb11.02.1720
Nv_IMB1	Nemve1_182805	Hs_XPO2	sp_P55060-1_XPO2_HUMAN
At_IMB1	tr_Q9FJD4_Q9FJD4_ARATH	Nv_XPO2	Nemve1_185581
Pp_IMB1	Phypa1_1_193928	At_XPO2	sp_Q9ZPY7_XPO2_ARATH
Ps_IMB1	Physo1_1_129795	Pp_XPO2	Phypa1_1_182124
Pr_IMB1	Phyra1_1_73238	Ps_XPO2	Physo1_1_134104
Lm_IMB1	LmjF34.0490	Pr_XPO2	Phyra1_1_81342
Tb_IMB1	Tb10.70.4720	Lm_XPO2	LmjF30.3390
Hs_IMB2	sp_Q92973_TNPO1_HUMAN	Tb_XPO2	Tb927.6.4740
Nv_IMB2	Nemve1_243273	Hs_TNPO3	sp_Q9Y5L0-1_TNPO3_HUMAN
At_IMB2	tr_Q8H2D6_Q8H2D6_ARATH	Nv_TNPO3	Nemve1_174976
Pp_IMB2	Phypa1_1_224057	At_TNPO3	tr_Q8GUL2_Q8GUL2_ARATH
Ps_IMB2	Physo1_1_109625	Pp_TNPO3	Phypa1_1_163909
Pr_IMB2	Phyra1_1_72223	Ps_TNPO3	Physo1_1_133433
Lm_IMB2	LmjF35.2330	Pr_TNPO3	Phyra1_1_80755
Tb_IMB2	Tb09.211.4360	Hs_XPO5	sp_Q9HAV4-1_XPO5_HUMAN
Hs_IMB3	tr_Q86XC7_Q86XC7_HUMAN	At_XPO5	tr_Q0WP44_Q0WP44_ARATH
Nv_IMB3	Nemve1_235624	Pp_XPO5	Phypa1_1_137344
At_IMB3	tr_Q93VS8_Q93VS8_ARATH	Ps_XPO5	Physo1_1_140752
Pp_IMB3	Phypa1_1_169387	Pr_XPO5	Phyra1_1_81240
Ps_IMB3	Physo1_1_139352	Hs_XPOT	sp_O43592_XPOT_HUMAN
Pr_IMB3	Phyra1_1_78209	Nv_XPOT	Nemve1_241098
Lm_IMB3	LmjF32.2150	At_XPOT	tr_Q7PC79_Q7PC79_ARATH
Tb_IMB3	Tb11.01.7010	Pp_XPOT	Phypa1_1_134096
Hs_IMB4	sp_Q8TEX9-2_IPO4_HUMAN	Hs_XPO1	sp_O14980_XPO1_HUMAN
Nv_IMB4	Nemve1_248390	Nv_XPO1	Nemve1_182854
At_IMB4	tr_Q8W498_Q8W498_ARATH	At_XPO1	tr_Q94IV0_Q94IV0_ARATH
Pp_IMB4	Phypa1_1_217532	Pp_XPO1	Phypa1_1_163030
Ps_IMB4	Physo1_1_134803	Ps_XPO1	Physo1_1_109057
Pr_IMB4	Phyra1_1_81386	Pr_XPO1	Phyra1_1_72043
Lm_IMB4	LmjF07.0720	Lm_XPO1	LmjF32.1100
Tb_IMB4	Tb11.01.8030	Tb_XPO1	Tb11.01.5940
Hs_IMB5	sp_Q96P70_IPO9_HUMAN	Hs_XPO4	sp_Q9C0E2_XPO4_HUMAN
Nv_IMB5	Nemve1_106835	At_XPO4	tr_Q9M838_Q9M838_ARATH
At_IMB5	tr_Q9C662_Q9C662_ARATH	Pp_XPO4	Phypa1_1_148515
Pp_IMB5	Phypa1_1_125599	Ps_XPO4	Physo1_1_143710
Ps_IMB5	Physo1_1_143359	Pr_XPO4	Phyra1_1_77078
Pr_IMB5	Phyra1_1_72932	Hs_XPO7	sp_Q9UIA9_XPO7_HUMAN
Hs_KA120	sp_Q9UI26_IPO11_HUMAN	Nv_XPO7	Nemve1_30364
Nv_KA120	Nemve1_248196	At_XPO7	tr_Q9LHS4_Q9LHS4_ARATH
At_KA120	tr_Q9SR95_Q9SR95_ARATH	Pp_XPO7	Phypa1_1_154305
Pp_KA120	Phypa1_1_152314	Ps_XPO7	Physo1_1_141370
Ps_KA120	Physo1_1_142102	Pr_XPO7	Phyra1_1_74843
Pr_KA120	Phyra1_1_76869	Lm_XPO7a	LmjF26.1900
Hs_IPO8	sp_O15397_IPO8_HUMAN	Tb_XPO7a	Tb09.160.1250
Nv_IPO8	Nemve1_177781	Lm_XPO7b	LmjF10.1200
At_IPO8	tr_Q27GK2_Q27GK2_ARATH	Tb_XPO7b	Tb927.8.4660

(a)
Three supergroups, all 15 subfamiles.

Conclusions:
XPO4 & XPO7 are derived from a common ancestor.
(IMB1, IMB2, IMB3, IMB4) and (IMB5, KA120, IPO8, XPO2) form clades and are derived from a common ancestor (full branch order not established)

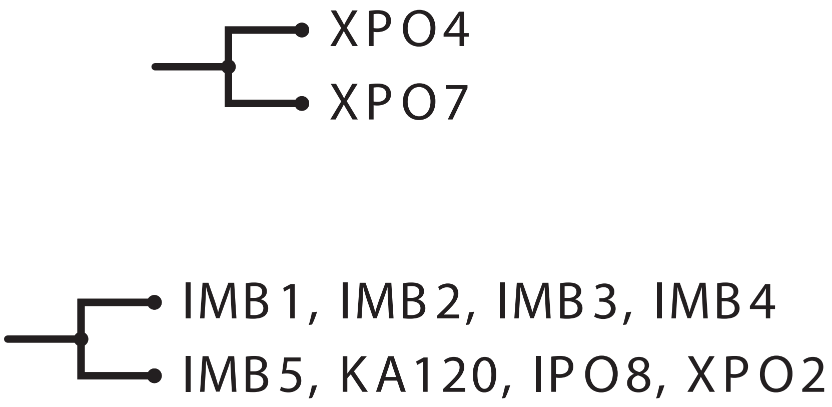

(a) MrBayes

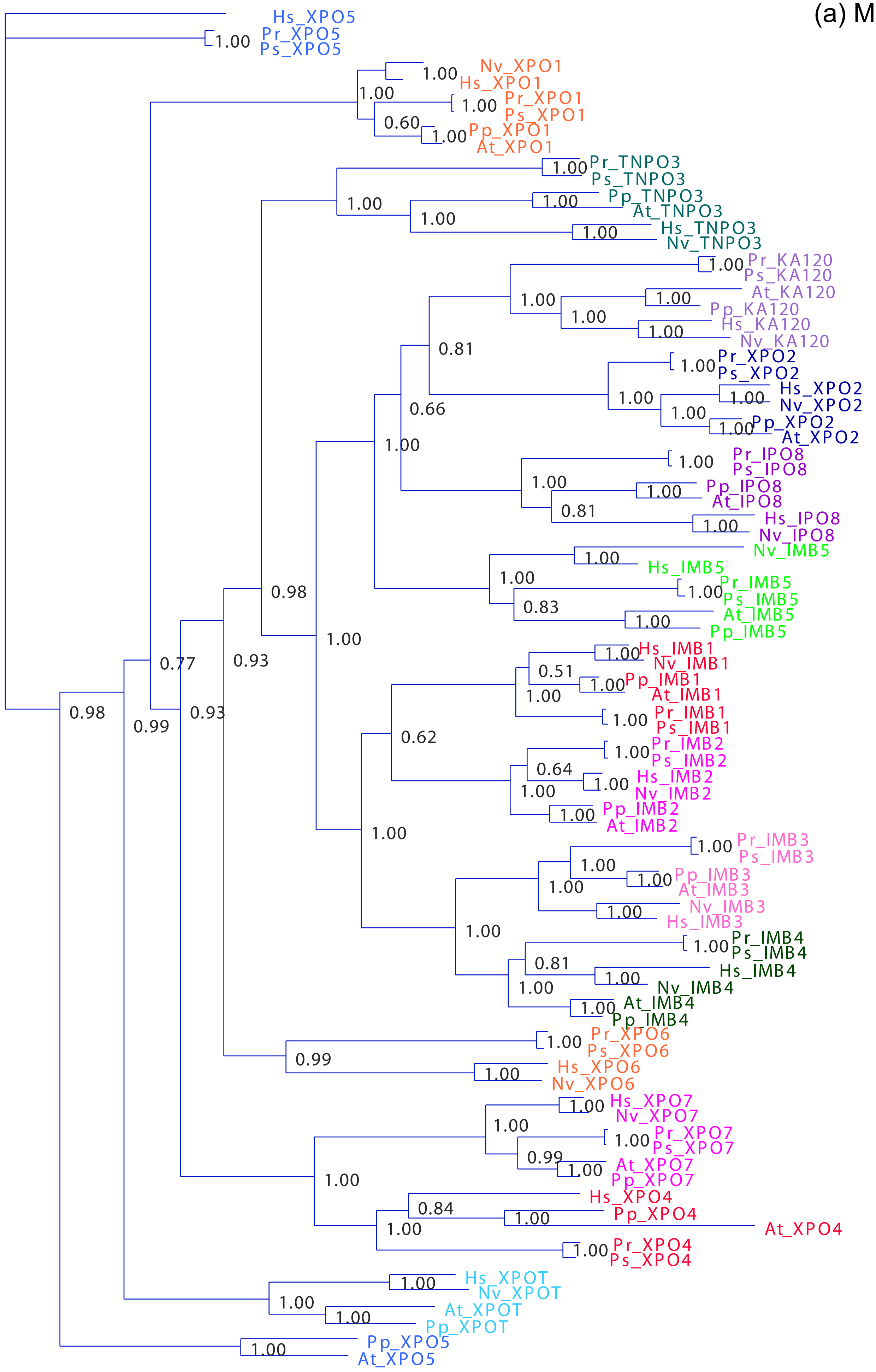
Hs_XPO5
Pr_XPO5
Ps_XPO5
Nv_XPO1
Hs_XPO1
Pr_XPO1
Ps_XPO1
Pp_XPO1
At_XPO1
Pr_TNPO3
Ps_TNPO3
Pp_TNPO3
At_TNPO3
Hs_TNPO3
Nv_TNPO3
Pr_KA120
Ps_KA120
At_KA120
Pp_KA120
Hs_KA120
Nv_KA120
Pr_XPO2
Ps_XPO2
Hs_XPO2
Nv_XPO2
Pp_XPO2
At_XPO2
Pr_IPO8
Ps_IPO8
Pp_IPO8
At_IPO8
Hs_IPO8
Nv_IPO8
Nv_IMB5
Hs_IMB5
Pr_IMB5
Ps_IMB5
At_IMB5
Pp_IMB5
Hs_IMB1
Nv_IMB1
Pp_IMB1
At_IMB1
Pr_IMB1
Ps_IMB1
Pr_IMB2
Ps_IMB2
Hs_IMB2
Nv_IMB2
Pp_IMB2
At_IMB2
Pr_IMB3
Ps_IMB3
Pp_IMB3
At_IMB3
Nv_IMB3
Hs_IMB3
Pr_IMB4
Ps_IMB4
Hs_IMB4
Nv_IMB4
At_IMB4
Pp_IMB4
Pr_XPO6
Ps_XPO6
Hs_XPO6
Nv_XPO6
Hs_XPO7
Nv_XPO7
Pr_XPO7
Ps_XPO7
At_XPO7
Pp_XPO7
Hs_XPO4
Pp_XPO4
At_XPO4
Pr_XPO4
Ps_XPO4
Hs_XPOT
Nv_XPOT
At_XPOT
Pp_XPOT
Pp_XPO5
At_XPO5

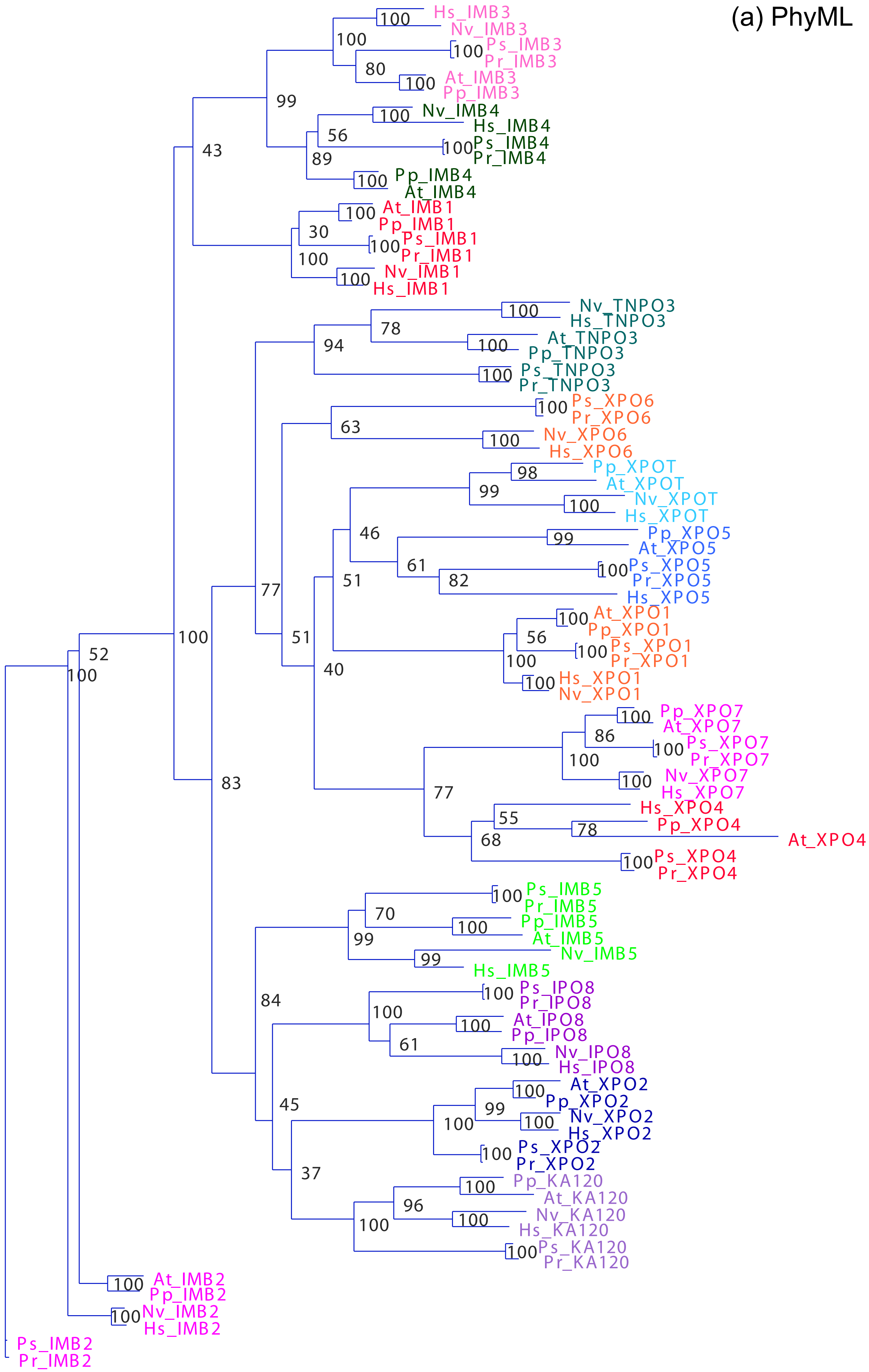
(a) PhyML
Hs_IMB3
Nv_IMB3
Ps_IMB3
Pr_IMB3
At_IMB3
Pp_IMB3
Nv_IMB4
Hs_IMB4
Ps_IMB4
Pr_IMB4
Pp_IMB4
At_IMB4
At_IMB1
Pp_IMB1
Ps_IMB1
Pr_IMB1
Nv_IMB1
Hs_IMB1
Nv_TNPO3
Hs_TNPO3
At_TNPO3
Pp_TNPO3
Ps_TNPO3
Pr_TNPO3
Ps_XPO6
Pr_XPO6
Nv_XPO6
Hs_XPO6
Pp_XPOT
At_XPOT
Nv_XPOT
Hs_XPOT
Pp_XPO5
At_XPO5
Ps_XPO5
Pr_XPO5
Hs_XPO5
At_XPO1
Pp_XPO1
Ps_XPO1
Pr_XPO1
Hs_XPO1
Nv_XPO1
Pp_XPO7
At_XPO7
Ps_XPO7
Pr_XPO7
Nv_XPO7
Hs_XPO7
Hs_XPO4
Pp_XPO4
At_XPO4
Ps_XPO4
Pr_XPO4
Ps_IMB5
Pr_IMB5
Pp_IMB5
At_IMB5
Nv_IMB5
Hs_IMB5
Ps_IPO8
Pr_IPO8
At_IPO8
Pp_IPO8
Nv_IPO8
Hs_IPO8
At_XPO2
Pp_XPO2
Nv_XPO2
Hs_XPO2
Ps_XPO2
Pr_XPO2
Pp_KA120
At_KA120
Nv_KA120
Hs_KA120
Ps_KA120
Pr_KA120
At_IMB2
Pp_IMB2
Nv_IMB2
Hs_IMB2
Ps_IMB2
Pr_IMB2

(b)
The subfamily representation in (a) was split in order to investigate branching order and ancestral relationships between family members.

Four supergroups, selected subfamilies.
(IMB1, IMB2, IMB3, IMB4, IMB5, KA120, IPO8, XPO2) only.

Conclusion:
branch order for IMB1, IMB2, IMB3, IMB4, IMB5, KA120, IPO8, XPO2 established.

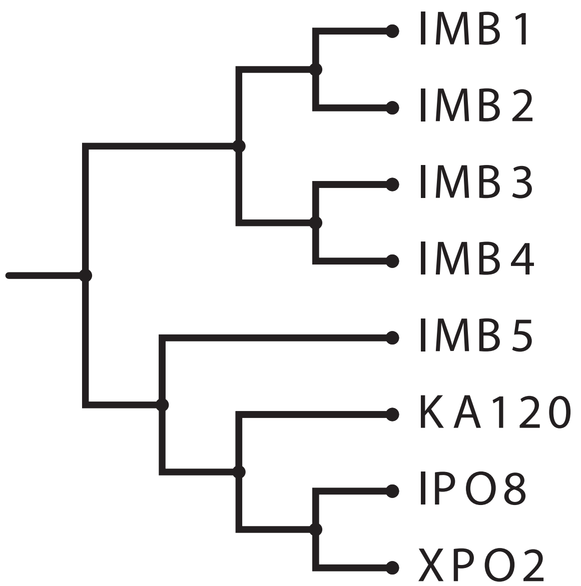

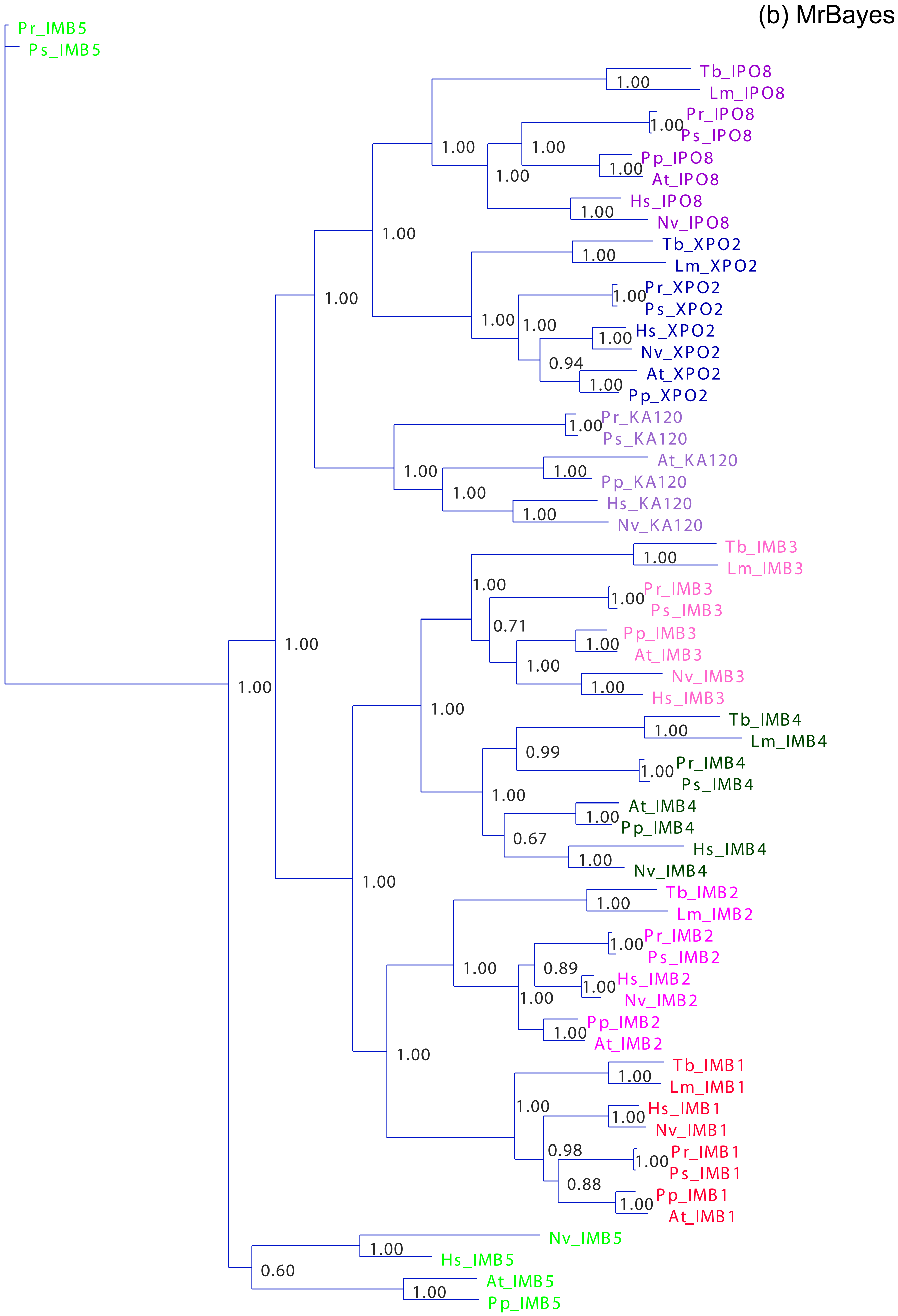
(b) MrBayes
Pr_IMB5
Ps_IMB5
Tb_IPO8
Lm_IPO8
Pr_IPO8
Ps_IPO8
Pp_IPO8
At_IPO8
Hs_IPO8
Nv_IPO8
Tb_XPO2
Lm_XPO2
Pr_XPO2
Ps_XPO2
Hs_XPO2
Nv_XPO2
At_XPO2
Pp_XPO2
Pr_KA120
Ps_KA120
At_KA120
Pp_KA120
Hs_KA120
Nv_KA120
Tb_IMB3
Lm_IMB3
Pr_IMB3
Ps_IMB3
Pp_IMB3
At_IMB3
Nv_IMB3
Hs_IMB3
Tb_IMB4
Lm_IMB4
Pr_IMB4
Ps_IMB4
At_IMB4
Pp_IMB4
Hs_IMB4
Nv_IMB4
Tb_IMB2
Lm_IMB2
Pr_IMB2
Ps_IMB2
Hs_IMB2
Nv_IMB2
Pp_IMB2
At_IMB2
Tb_IMB1
Lm_IMB1
Hs_IMB1
Nv_IMB1
Pr_IMB1
Ps_IMB1
Pp_IMB1
At_IMB1
Nv_IMB5
Hs_IMB5
At_IMB5
Pp_IMB5
0.94
0.71
0.99
0.67
0.89
0.98
0.88
0.60

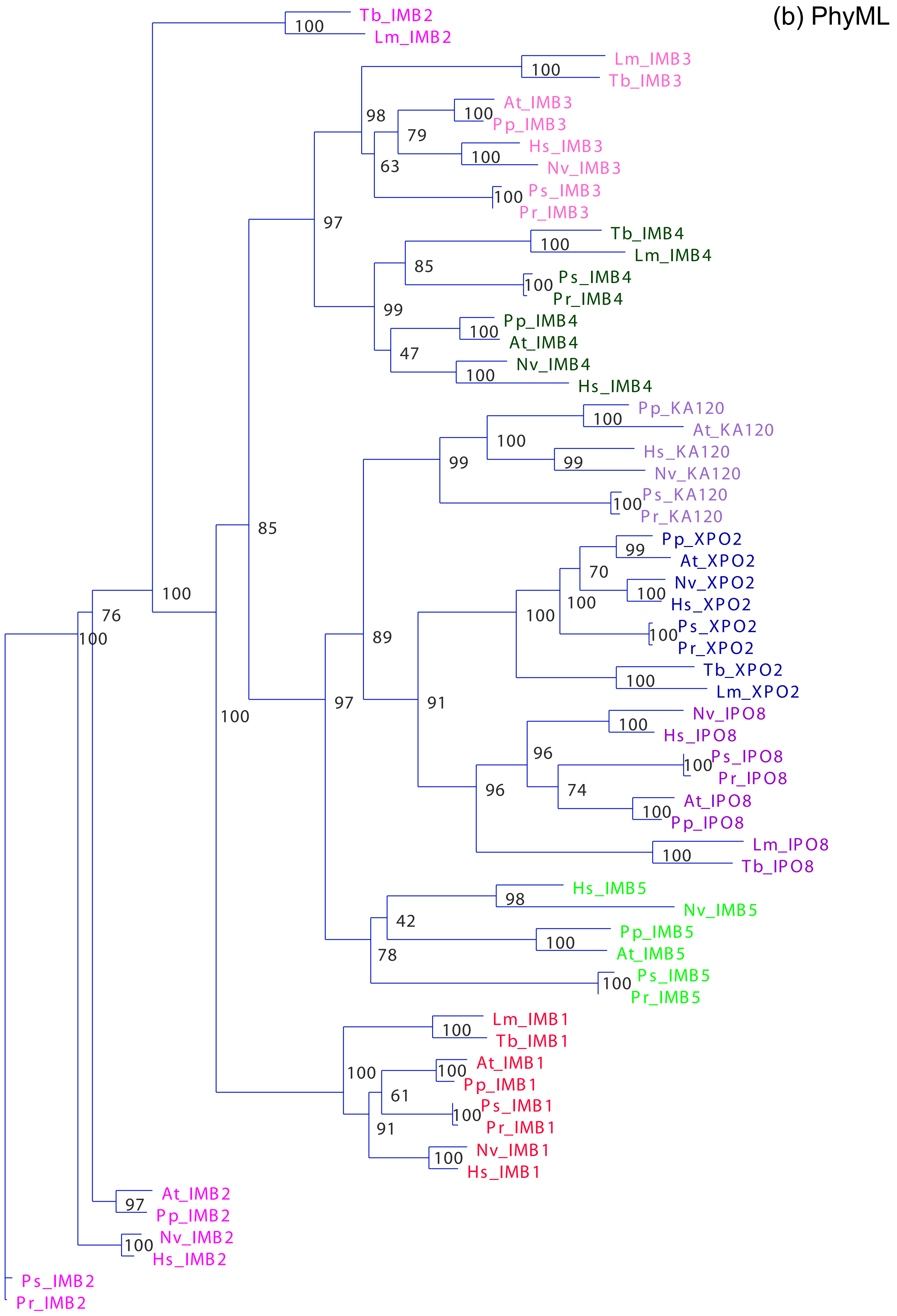
(b) PhyML
Tb_IMB2
Lm_IMB2
Lm_IMB3
Tb_IMB3
At_IMB3
Pp_IMB3
Hs_IMB3
Nv_IMB3
Ps_IMB3
Pr_IMB3
Tb_IMB4
Lm_IMB4
Ps_IMB4
Pr_IMB4
Pp_IMB4
At_IMB4
Nv_IMB4
Hs_IMB4
Pp_KA120
At_KA120
Hs_KA120
Nv_KA120
Ps_KA120
Pr_KA120
Pp_XPO2
At_XPO2
Nv_XPO2
Hs_XPO2
Ps_XPO2
Pr_XPO2
Tb_XPO2
Lm_XPO2
Nv_IPO8
Hs_IPO8
Ps_IPO8
Pr_IPO8
At_IPO8
Pp_IPO8
Lm_IPO8
Tb_IPO8
Hs_IMB5
Nv_IMB5
Pp_IMB5
At_IMB5
Ps_IMB5
Pr_IMB5
Lm_IMB1
Tb_IMB1
At_IMB1
Pp_IMB1
Ps_IMB1
Pr_IMB1
Nv_IMB1
Hs_IMB1
At_IMB2
Pp_IMB2
Nv_IMB2
Hs_IMB2
Ps_IMB2
Pr_IMB2

(c)
Three supergroups, all subfamilies not in (b)
(so the branch leading to the members of the (b) analysis is missing).

Conclusions:
XPO6 forms a clade with (XPO4, XPO7).
Branch order of (TNPO3, XPO5, XPOT, XPO1) not established.

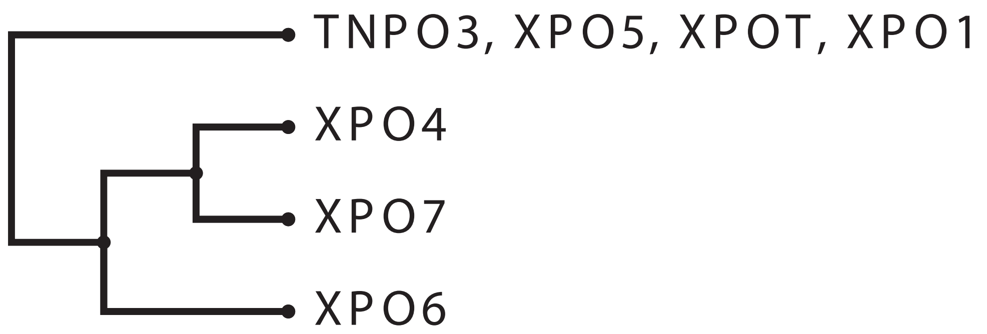

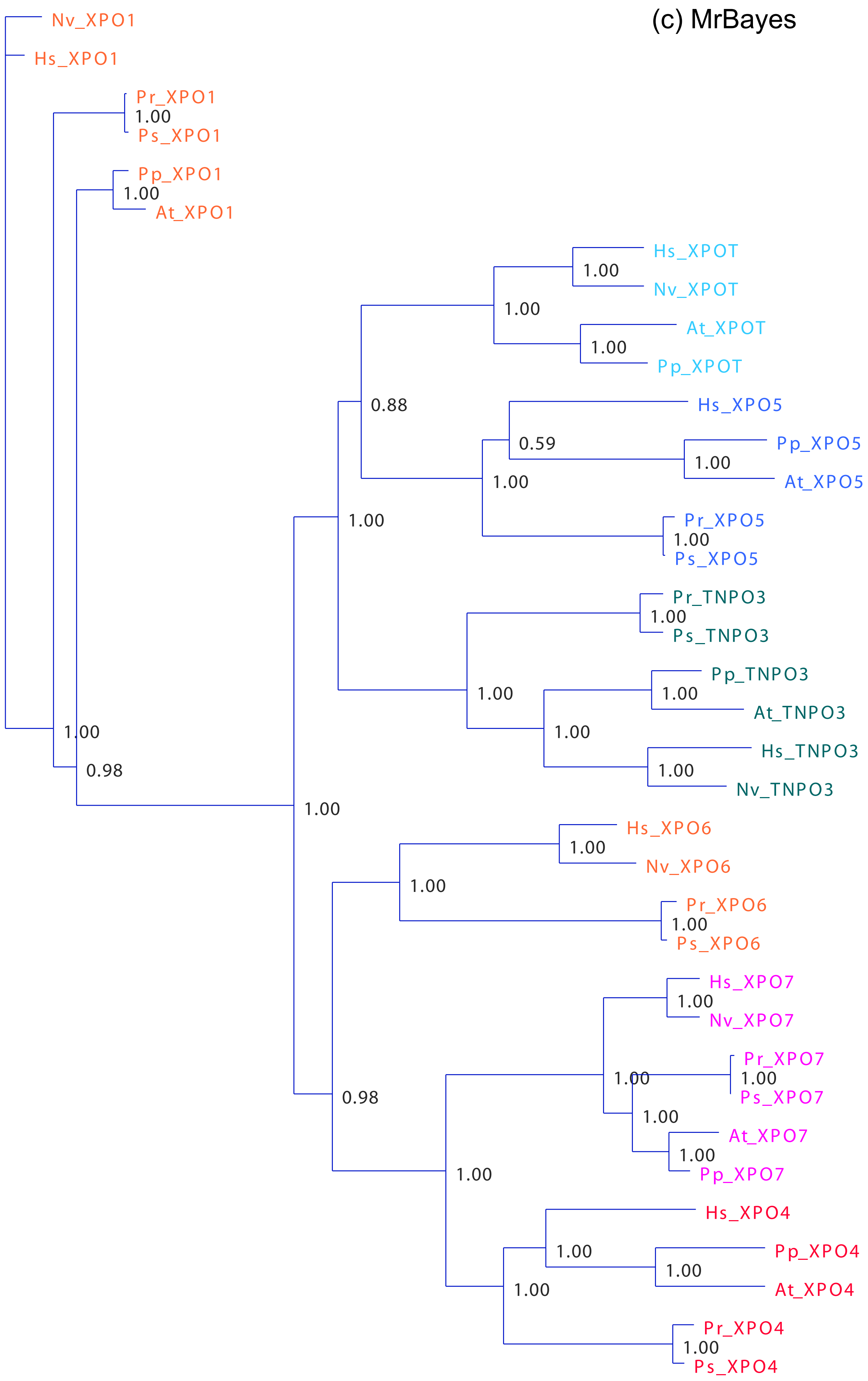
(c) MrBayes
Nv_XPO1
Hs_XPO1
Pr_XPO1
1.00
Ps_XPO1
Pp_XPO1
1.00
At_XPO1
Hs_XPOT
1.00
Nv_XPOT
1.00
At_XPOT
1.00
Pp_XPOT
0.88
Hs_XPO5
0.59
Pp_XPO5
1.00
At_XPO5
1.00
1.00
Pr_XPO5
1.00
Ps_XPO5
Pr_TNPO3
1.00
Ps_TNPO3
Pp_TNPO3
1.00
1.00
At_TNPO3
1.00
Hs_TNPO3
1.00
Nv_TNPO3
1.00
0.98
1.00
Hs_XPO6
1.00
Nv_XPO6
1.00
Pr_XPO6
1.00
Ps_XPO6
Hs_XPO7
1.00
Nv_XPO7
Pr_XPO7
1.00
1.00
Ps_XPO7
0.98
1.00
At_XPO7
1.00
1.00
Pp_XPO7
Hs_XPO4
Pp_XPO4
1.00
1.00
At_XPO4
1.00
Pr_XPO4
1.00
Ps_XPO4

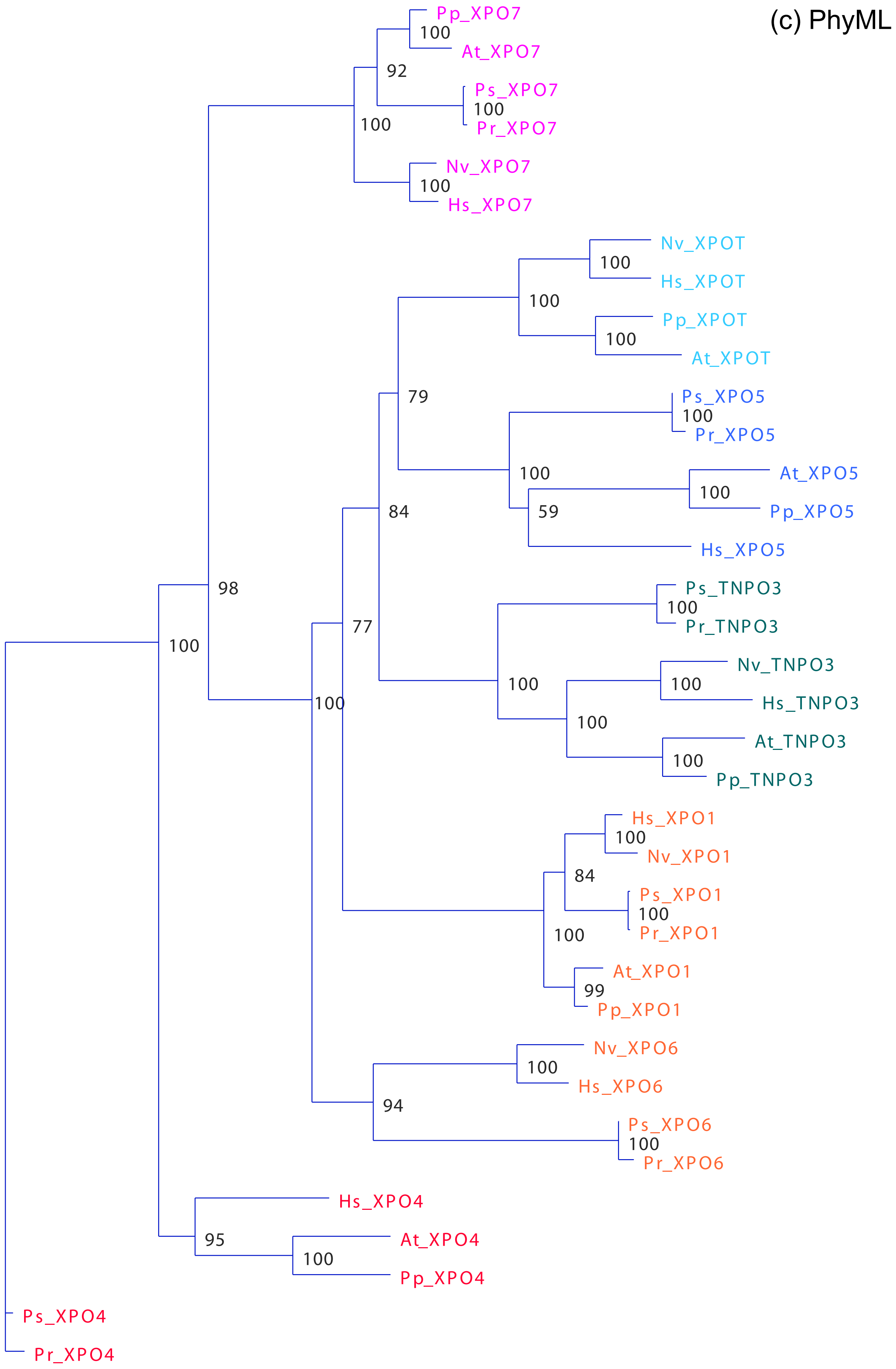
(c) PhyML
Pp_XPO7
At_XPO7
Ps_XPO7
Pr_XPO7
Nv_XPO7
Hs_XPO7
Nv_XPOT
Hs_XPOT
Pp_XPOT
At_XPOT
Ps_XPO5
Pr_XPO5
At_XPO5
Pp_XPO5
Hs_XPO5
Ps_TNPO3
Pr_TNPO3
Nv_TNPO3
Hs_TNPO3
At_TNPO3
Pp_TNPO3
Hs_XPO1
Nv_XPO1
Ps_XPO1
Pr_XPO1
At_XPO1
Pp_XPO1
Nv_XPO6
Hs_XPO6
Ps_XPO6
Pr_XPO6
Hs_XPO4
At_XPO4
Pp_XPO4
Ps_XPO4
Pr_XPO4

(d)
Removed XPO4 and XPO6 subfamilies from (c) in order to establish branching order in (TNPO3, XPO5, XPOT, XPO1) clade.

Three supergroups.

Conclusion:
branch order for TNPO3, XPO5, XPOT, XPO1 established

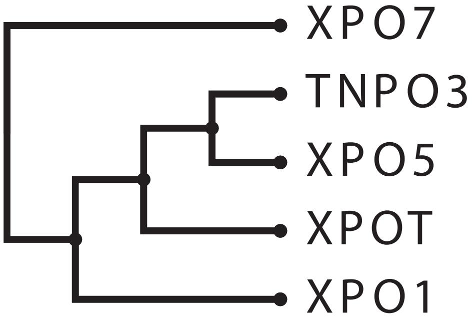

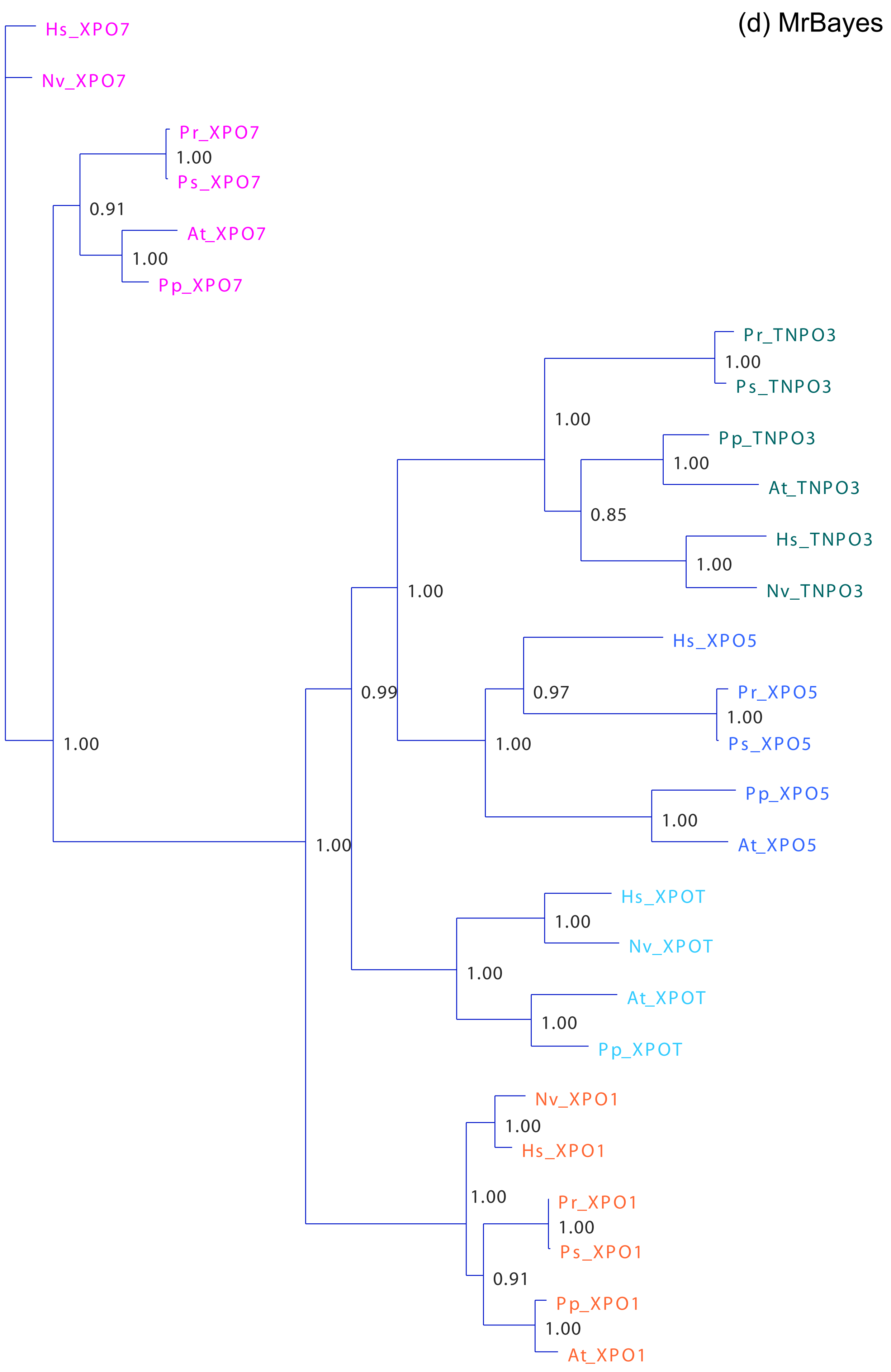
(d) MrBayes
Hs_XPO7
Nv_XPO7
Pr_XPO7
1.00
Ps_XPO7
0.91
At_XPO7
1.00
Pp_XPO7
Pr_TNPO3
1.00
Ps_TNPO3
1.00
Pp_TNPO3
1.00
At_TNPO3
0.85
Hs_TNPO3
1.00
Nv_TNPO3
1.00
Hs_XPO5
0.97
Pr_XPO5
1.00
Ps_XPO5
0.99
1.00
1.00
Pp_XPO5
1.00
At_XPO5
1.00
Hs_XPOT
1.00
Nv_XPOT
1.00
At_XPOT
1.00
Pp_XPOT
Nv_XPO1
1.00
Hs_XPO1
1.00
Pr_XPO1
1.00
Ps_XPO1
0.91
Pp_XPO1
1.00
At_XPO1

(d) PhyML

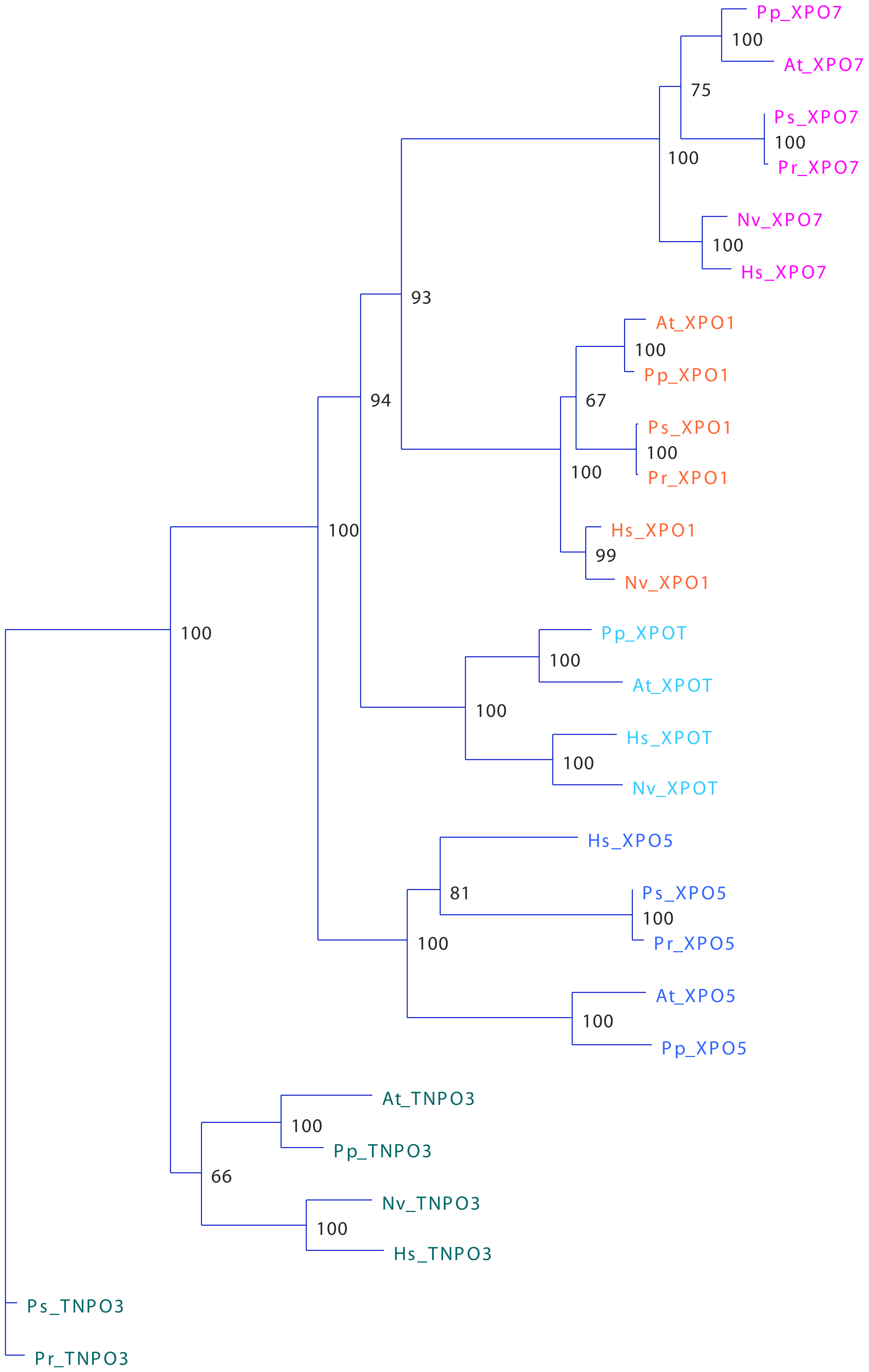
Pp_XPO7
100
At_XPO7
75
Ps_XPO7
100
Pr_XPO7
100
Nv_XPO7
100
Hs_XPO7
93
At_XPO1
100
Pp_XPO1
94
67
Ps_XPO1
100
Pr_XPO1
100
100
Hs_XPO1
99
Nv_XPO1
100
Pp_XPOT
100
At_XPOT
100
Hs_XPOT
100
Nv_XPOT
Hs_XPO5
81
Ps_XPO5
100
Pr_XPO5
100
At_XPO5
100
Pp_XPO5
At_TNPO3
100
Pp_TNPO3
66
Nv_TNPO3
100
Hs_TNPO3
Ps_TNPO3
Pr_TNPO3

(e)
Four supergroups, XPO1, XPO2, XPO6, XPO7 subfamilies.
XPO1 represents (TNPO3, XPO5, XPOT, XPO1) clade
XPO2 represents (IMB1, IMB2, IMB3, IMB4, IMB5, KA120, IPO8, XPO2) clade
XPO7 represents (XPO4, XPO7) clade

Conclusion:
branch order for XPO1, XPO2, XPO6, XPO7 established.

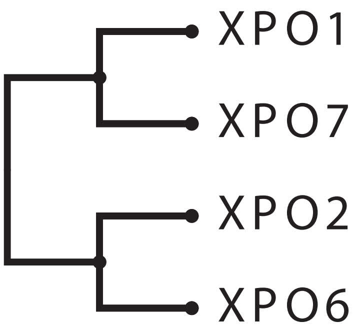

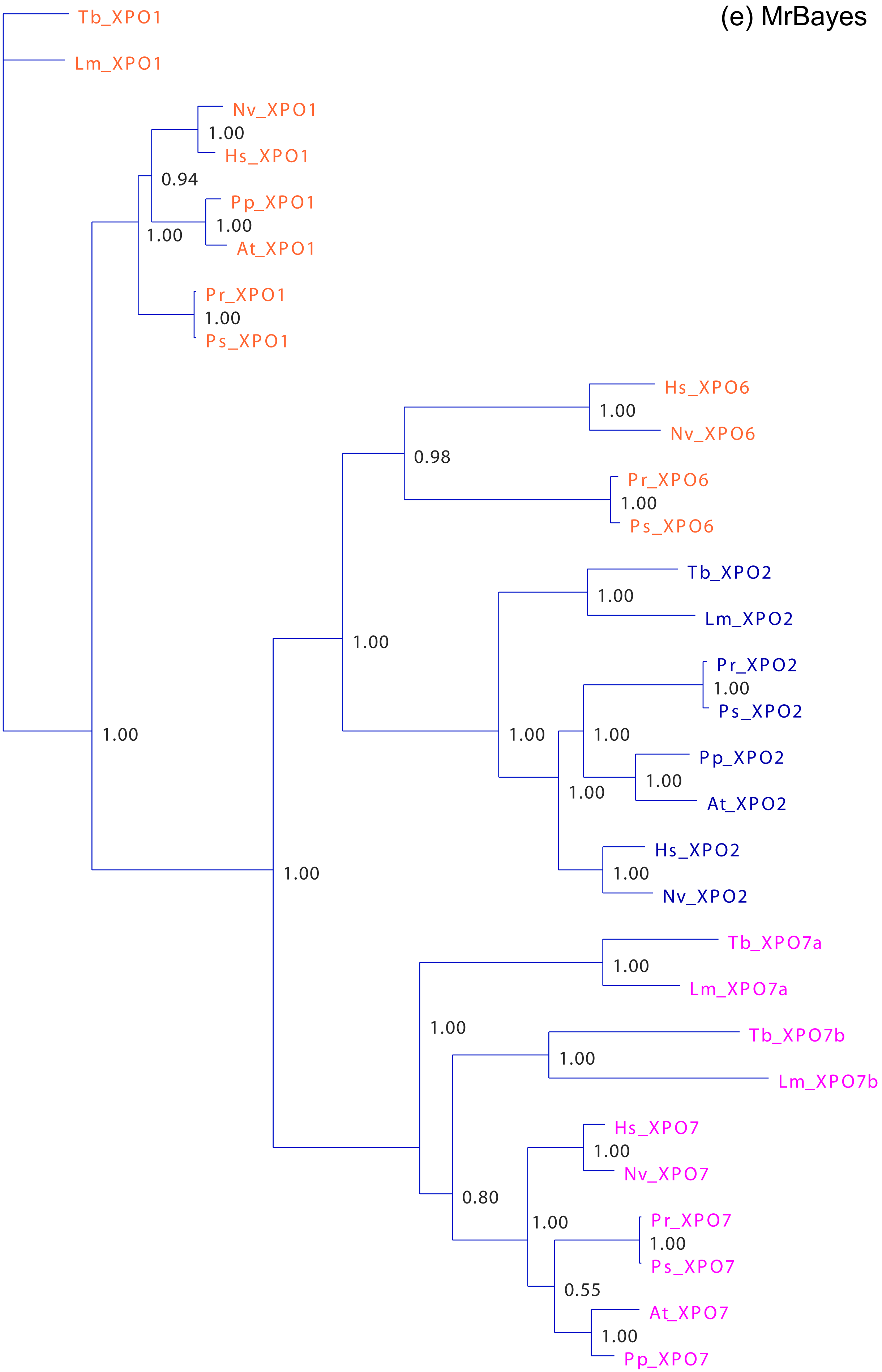
(e) MrBayes
Tb_XPO1
Lm_XPO1
Nv_XPO1
1.00
Hs_XPO1
0.94
Pp_XPO1
1.00
1.00
At_XPO1
Pr_XPO1
1.00
Ps_XPO1
Hs_XPO6
1.00
Nv_XPO6
0.98
Pr_XPO6
1.00
Ps_XPO6
Tb_XPO2
1.00
Lm_XPO2
1.00
Pr_XPO2
1.00
Ps_XPO2
1.00
1.00
1.00
Pp_XPO2
1.00
1.00
At_XPO2
Hs_XPO2
1.00
Nv_XPO2
1.00
Tb_XPO7a
1.00
Lm_XPO7a
1.00
Tb_XPO7b
1.00
Lm_XPO7b
Hs_XPO7
1.00
Nv_XPO7
0.80
1.00
Pr_XPO7
1.00
Ps_XPO7
0.55
At_XPO7
1.00
Pp_XPO7

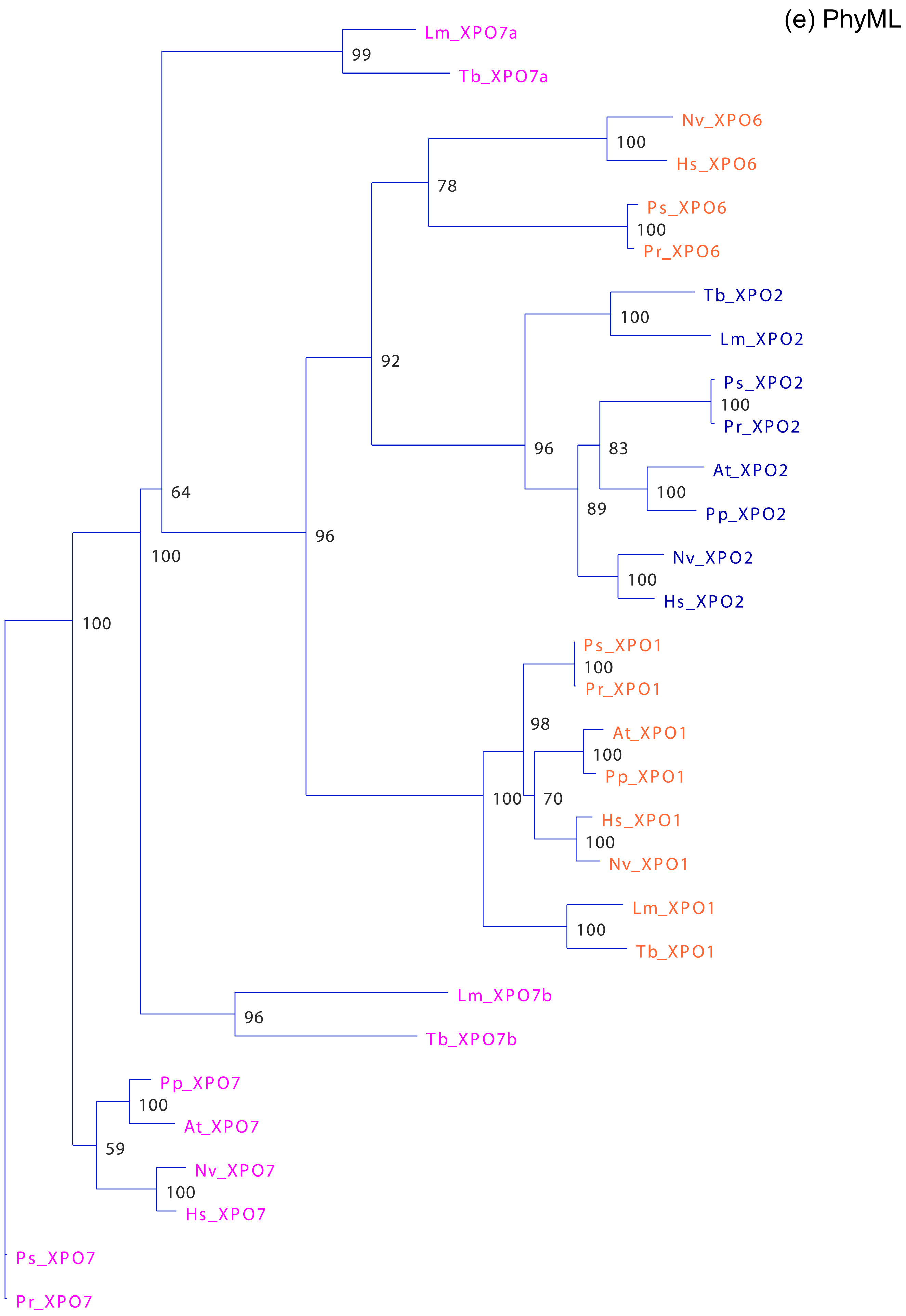
(e) PhyML
Lm_XPO7a
99
Tb_XPO7a
Nv_XPO6
100
Hs_XPO6
78
Ps_XPO6
100
Pr_XPO6
Tb_XPO2
100
Lm_XPO2
92
Ps_XPO2
100
Pr_XPO2
96
83
At_XPO2
100
Pp_XPO2
89
64
Nv_XPO2
100
Hs_XPO2
96
100
100
Ps_XPO1
100
Pr_XPO1
98
At_XPO1
100
Pp_XPO1
100
70
Hs_XPO1
100
Nv_XPO1
Lm_XPO1
100
Tb_XPO1
Lm_XPO7b
96
Tb_XPO7b
Pp_XPO7
100
At_XPO7
59
Nv_XPO7
100
Hs_XPO7
Ps_XPO7
Pr_XPO7
