# Supplementary material for: Evolution of the Karyopherin-β Family of Nucleocytoplasmic Transport Factors; Ancient Origins and Continued Specialization
Source: PLoS One. 2011 Apr 27;6(4):e19308. doi: 10.1371/journal.pone.0019308 (PMC3083441; doi:10.1371/journal.pone.0019308)
Supplement: Figure S2 — Phylogenetic trees constructed using MrBayes showing lineage-specific expansions and NJ tree containing all TNPO3 candidate sequences, see main text for details. (PDF) [file pone.0019308.s002.pdf]

Figure S2

(a-g) MrBayes trees showing lineage-specific events indicated with arrows.
Subfamily names have been added to each taxon label as "__NAME".

(h) ClustalW neighbour-joining tree of TNPO3 candidates.

Species	ID key:
HUMAN	*Homo sapiens*
DANRE	*Danio rerio*
Nemve	*Nematostella vectensis*
DICDI	*Dictyostelium discoideum* (strain AX4)
ARATH	*Arabidopsis thaliana* (cultivar Columbia)
Poptr	*Populus trichocarpa*
ORYSJ	*Oryza sativa* (subsp. japonica, cultivar Japonica, strain Nipponbare)
Phypa	*Physcomitrella patens* ssp patens ecotype Gransden 2004
Physo	*Phytophthora sojae*
Phyra	*Phytophthora ramorum*
Lm	*Leishmania major*
Tb	*Trypanosoma brucei*
Tc	*Trypanosoma cruzi*

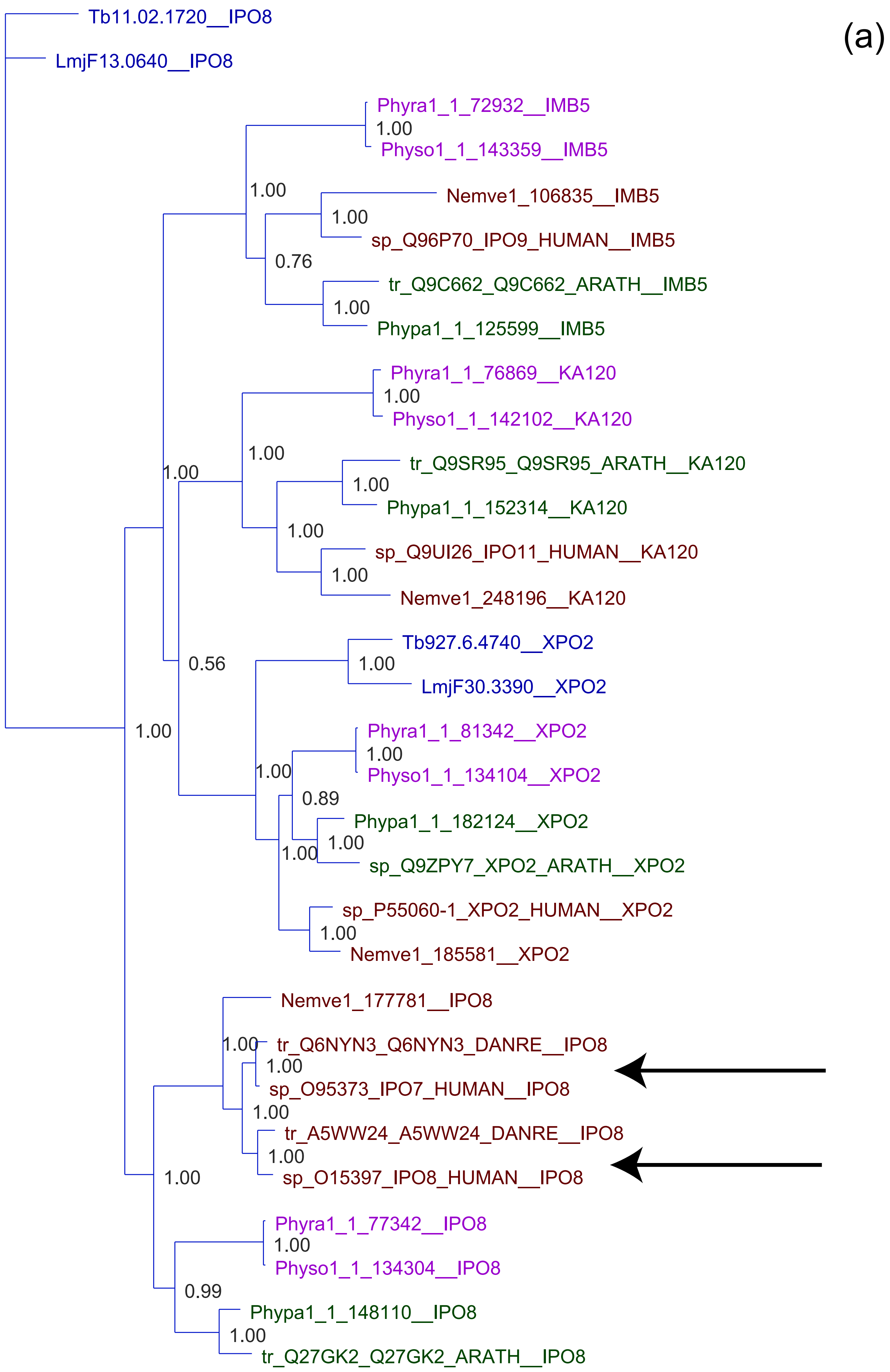

(a)
Tb11.02.1720__IPO8
LmjF13.0640__IPO8
Phyra1_1_72932__IMB5
1.00
Physo1_1_143359__IMB5
1.00
Nemve1_106835__IMB5
1.00
sp_Q96P70_IPO9_HUMAN__IMB5
0.76
tr_Q9C662_Q9C662_ARATH__IMB5
1.00
Phypa1_1_125599__IMB5
Phyra1_1_76869__KA120
1.00
Physo1_1_142102__KA120
1.00
1.00
tr_Q9SR95_Q9SR95_ARATH__KA120
1.00
Phypa1_1_152314__KA120
1.00
sp_Q9UI26_IPO11_HUMAN__KA120
1.00
Nemve1_248196__KA120
Tb927.6.4740__XPO2
0.56
1.00
LmjF30.3390__XPO2
1.00
Phyra1_1_81342__XPO2
1.00
Physo1_1_134104__XPO2
1.00
0.89
Phypa1_1_182124__XPO2
1.00
1.00
sp_Q9ZPY7_XPO2_ARATH__XPO2
sp_P55060-1_XPO2_HUMAN__XPO2
1.00
Nemve1_185581__XPO2
Nemve1_177781__IPO8
1.00
tr_Q6NYN3_Q6NYN3_DANRE__IPO8
1.00
sp_O95373_IPO7_HUMAN__IPO8
1.00
tr_A5WW24_A5WW24_DANRE__IPO8
1.00
sp_O15397_IPO8_HUMAN__IPO8
1.00
Phyra1_1_77342__IPO8
1.00
Physo1_1_134304__IPO8
0.99
Phypa1_1_148110__IPO8
1.00
tr_Q27GK2_Q27GK2_ARATH__IPO8

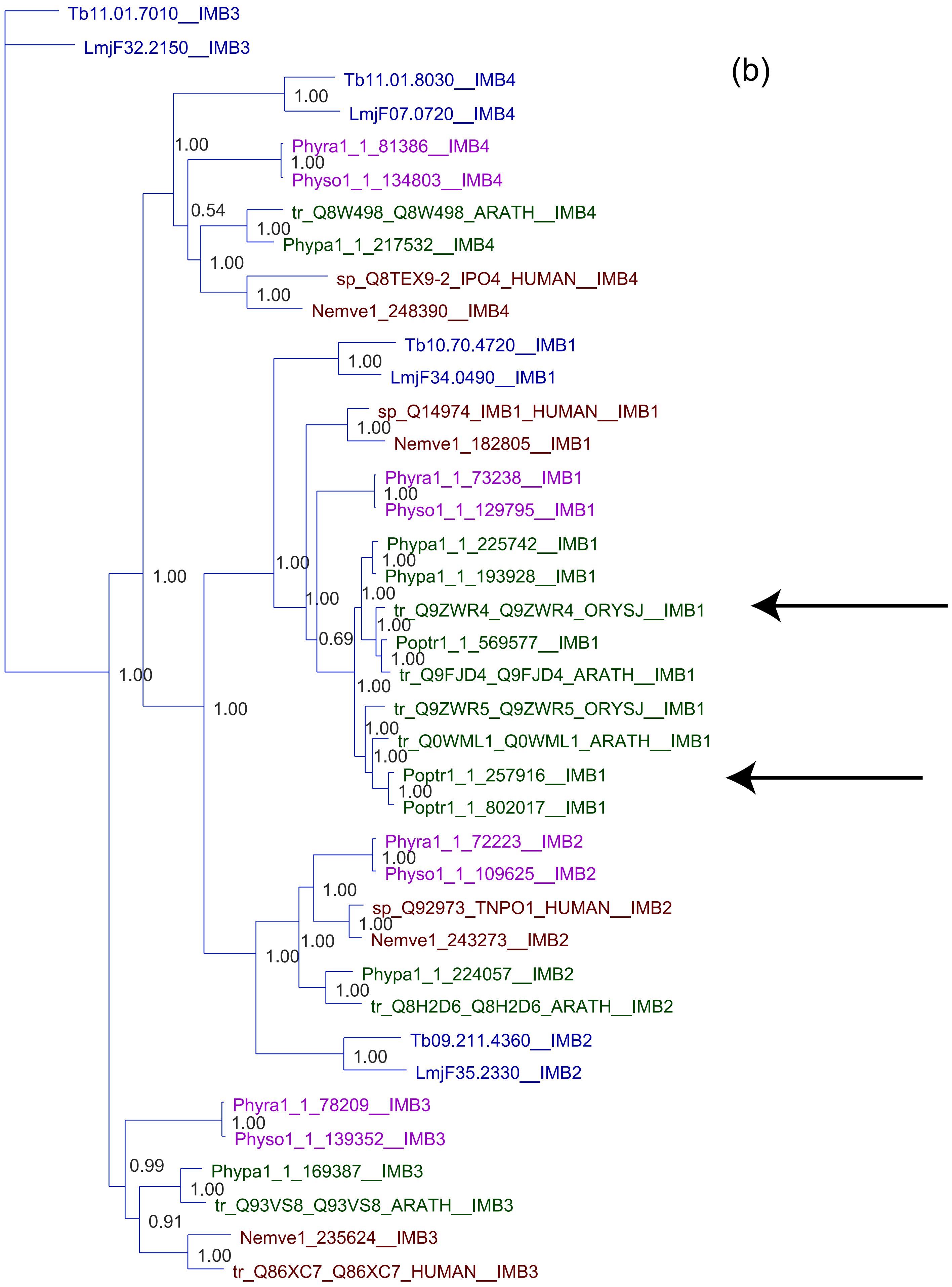
(b)
Tb11.01.7010__IMB3
LmjF32.2150__IMB3
Tb11.01.8030__IMB4
LmjF07.0720__IMB4
Phyra1_1_81386__IMB4
Physo1_1_134803__IMB4
tr_Q8W498_Q8W498_ARATH__IMB4
Phypa1_1_217532__IMB4
sp_Q8TEX9-2_IPO4_HUMAN__IMB4
Nemve1_248390__IMB4
Tb10.70.4720__IMB1
LmjF34.0490__IMB1
sp_Q14974_IMB1_HUMAN__IMB1
Nemve1_182805__IMB1
Phyra1_1_73238__IMB1
Physo1_1_129795__IMB1
Phypa1_1_225742__IMB1
Phypa1_1_193928__IMB1
tr_Q9ZWR4_Q9ZWR4_ORYSJ__IMB1
Poptr1_1_569577__IMB1
tr_Q9FJD4_Q9FJD4_ARATH__IMB1
tr_Q9ZWR5_Q9ZWR5_ORYSJ__IMB1
tr_Q0WML1_Q0WML1_ARATH__IMB1
Poptr1_1_257916__IMB1
Poptr1_1_802017__IMB1
Phyra1_1_72223__IMB2
Physo1_1_109625__IMB2
sp_Q92973_TNPO1_HUMAN__IMB2
Nemve1_243273__IMB2
Phypa1_1_224057__IMB2
tr_Q8H2D6_Q8H2D6_ARATH__IMB2
Tb09.211.4360__IMB2
LmjF35.2330__IMB2
Phyra1_1_78209__IMB3
Physo1_1_139352__IMB3
Phypa1_1_169387__IMB3
tr_Q93VS8_Q93VS8_ARATH__IMB3
Nemve1_235624__IMB3
tr_Q86XC7_Q86XC7_HUMAN__IMB3
0.54
0.69
0.99
0.91
1.00

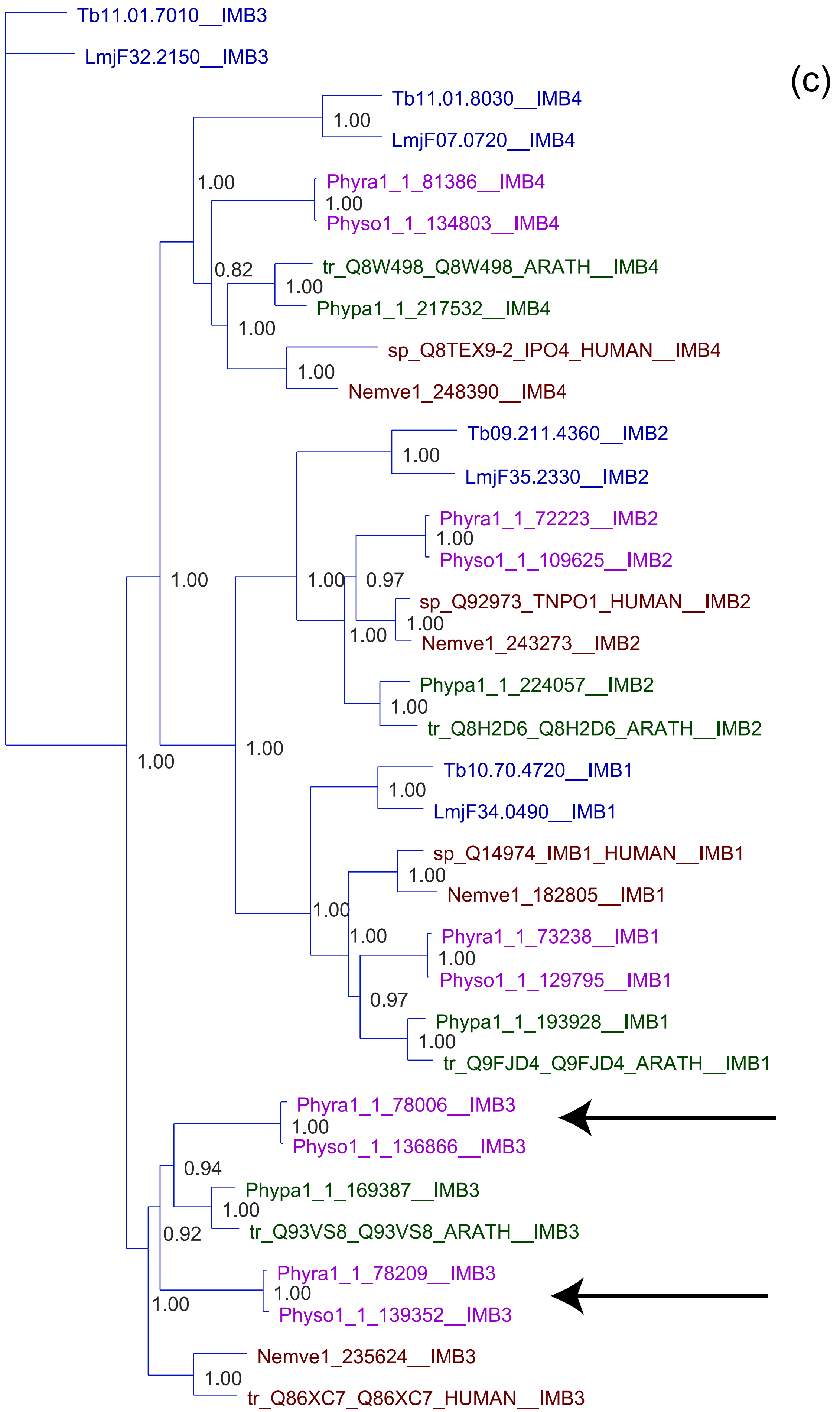
(c)
Tb11.01.7010__IMB3
LmjF32.2150__IMB3
Tb11.01.8030__IMB4
1.00
LmjF07.0720__IMB4
1.00
Phyra1_1_81386__IMB4
1.00
Physo1_1_134803__IMB4
0.82
tr_Q8W498_Q8W498_ARATH__IMB4
1.00
Phypa1_1_217532__IMB4
1.00
sp_Q8TEX9-2_IPO4_HUMAN__IMB4
1.00
Nemve1_248390__IMB4
Tb09.211.4360__IMB2
1.00
LmjF35.2330__IMB2
Phyra1_1_72223__IMB2
1.00
Physo1_1_109625__IMB2
1.00
1.00
0.97
sp_Q92973_TNPO1_HUMAN__IMB2
1.00
1.00
Nemve1_243273__IMB2
Phypa1_1_224057__IMB2
1.00
tr_Q8H2D6_Q8H2D6_ARATH__IMB2
1.00
1.00
Tb10.70.4720__IMB1
1.00
LmjF34.0490__IMB1
sp_Q14974_IMB1_HUMAN__IMB1
1.00
Nemve1_182805__IMB1
1.00
1.00
Phyra1_1_73238__IMB1
1.00
Physo1_1_129795__IMB1
0.97
Phypa1_1_193928__IMB1
1.00
tr_Q9FJD4_Q9FJD4_ARATH__IMB1
Phyra1_1_78006__IMB3
1.00
Physo1_1_136866__IMB3
0.94
Phypa1_1_169387__IMB3
1.00
0.92
tr_Q93VS8_Q93VS8_ARATH__IMB3
Phyra1_1_78209__IMB3
1.00
Physo1_1_139352__IMB3
1.00
Nemve1_235624__IMB3
1.00
tr_Q86XC7_Q86XC7_HUMAN__IMB3

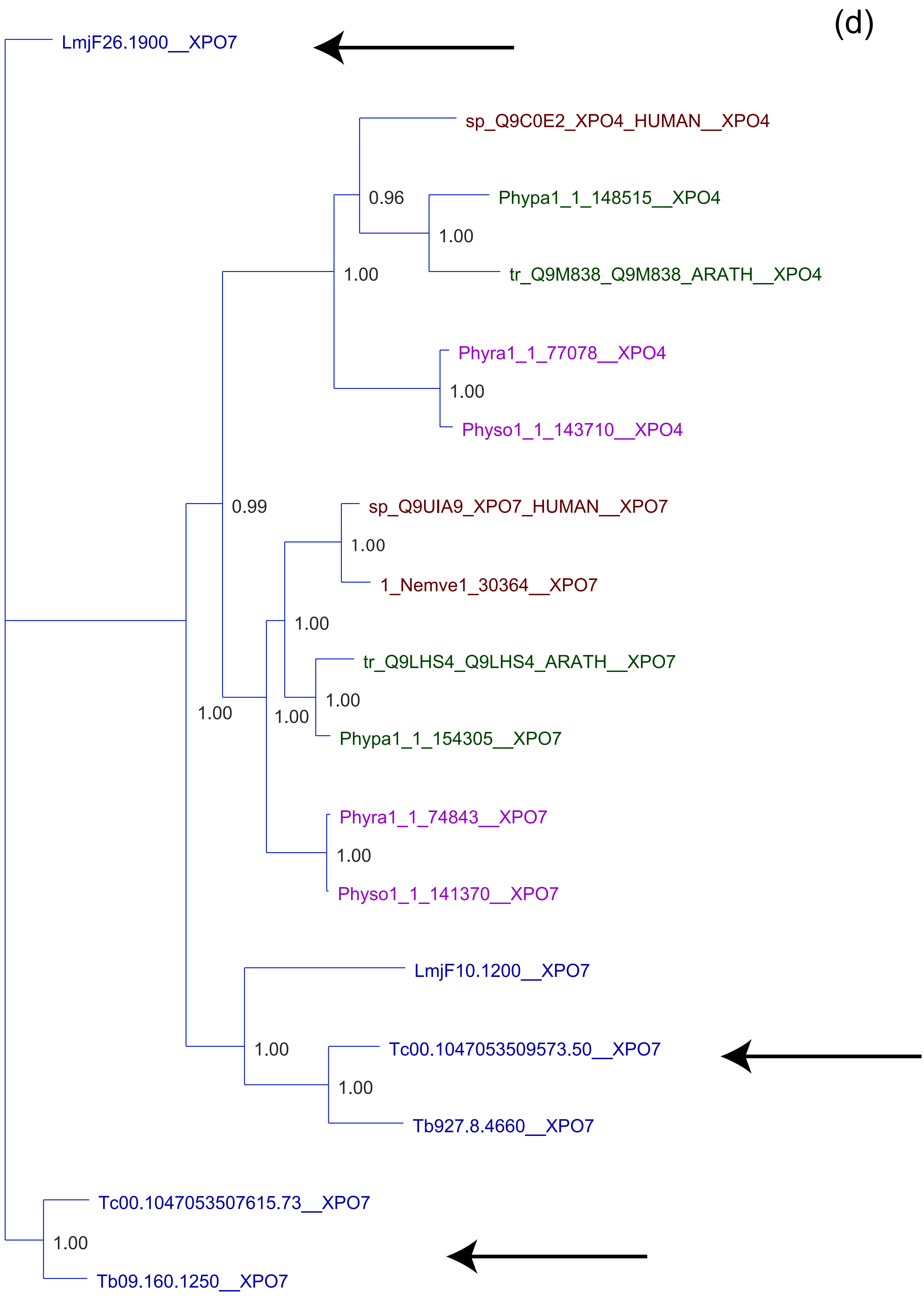
(d)
LmjF26.1900__XPO7
sp_Q9C0E2_XPO4_HUMAN__XPO4
0.96
Phypa1_1_148515__XPO4
1.00
tr_Q9M838_Q9M838_ARATH__XPO4
1.00
Phyra1_1_77078__XPO4
1.00
Physo1_1_143710__XPO4
0.99
sp_Q9UIA9_XPO7_HUMAN__XPO7
1.00
1_Nemve1_30364__XPO7
1.00
tr_Q9LHS4_Q9LHS4_ARATH__XPO7
1.00
1.00
1.00
Phypa1_1_154305__XPO7
Phyra1_1_74843__XPO7
1.00
Physo1_1_141370__XPO7
LmjF10.1200__XPO7
1.00
Tc00.1047053509573.50__XPO7
1.00
Tb927.8.4660__XPO7
Tc00.1047053507615.73__XPO7
1.00
Tb09.160.1250__XPO7

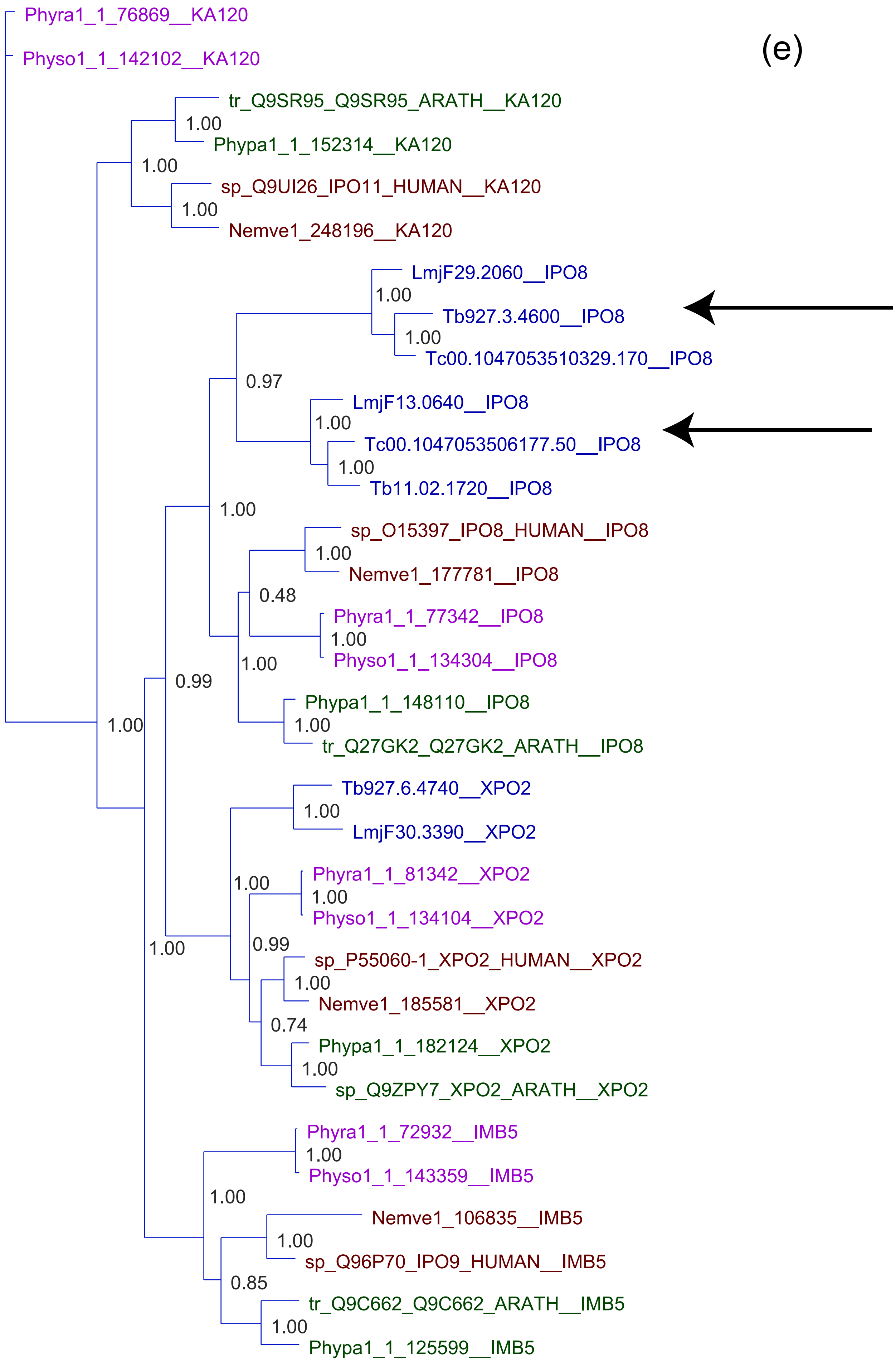
(e)
Phyra1_1_76869__KA120
Physo1_1_142102__KA120
tr_Q9SR95_Q9SR95_ARATH__KA120
1.00
Phypa1_1_152314__KA120
1.00
sp_Q9UI26_IPO11_HUMAN__KA120
1.00
Nemve1_248196__KA120
LmjF29.2060__IPO8
1.00
Tb927.3.4600__IPO8
1.00
Tc00.1047053510329.170__IPO8
0.97
LmjF13.0640__IPO8
1.00
Tc00.1047053506177.50__IPO8
1.00
Tb11.02.1720__IPO8
1.00
sp_O15397_IPO8_HUMAN__IPO8
1.00
Nemve1_177781__IPO8
0.48
Phyra1_1_77342__IPO8
1.00
Physo1_1_134304__IPO8
1.00
0.99
Phypa1_1_148110__IPO8
1.00
1.00
tr_Q27GK2_Q27GK2_ARATH__IPO8
Tb927.6.4740__XPO2
1.00
LmjF30.3390__XPO2
Phyra1_1_81342__XPO2
1.00
1.00
Physo1_1_134104__XPO2
1.00
0.99
sp_P55060-1_XPO2_HUMAN__XPO2
1.00
Nemve1_185581__XPO2
0.74
Phypa1_1_182124__XPO2
1.00
sp_Q9ZPY7_XPO2_ARATH__XPO2
Phyra1_1_72932__IMB5
1.00
Physo1_1_143359__IMB5
1.00
Nemve1_106835__IMB5
1.00
sp_Q96P70_IPO9_HUMAN__IMB5
0.85
tr_Q9C662_Q9C662_ARATH__IMB5
1.00
Phypa1_1_125599__IMB5

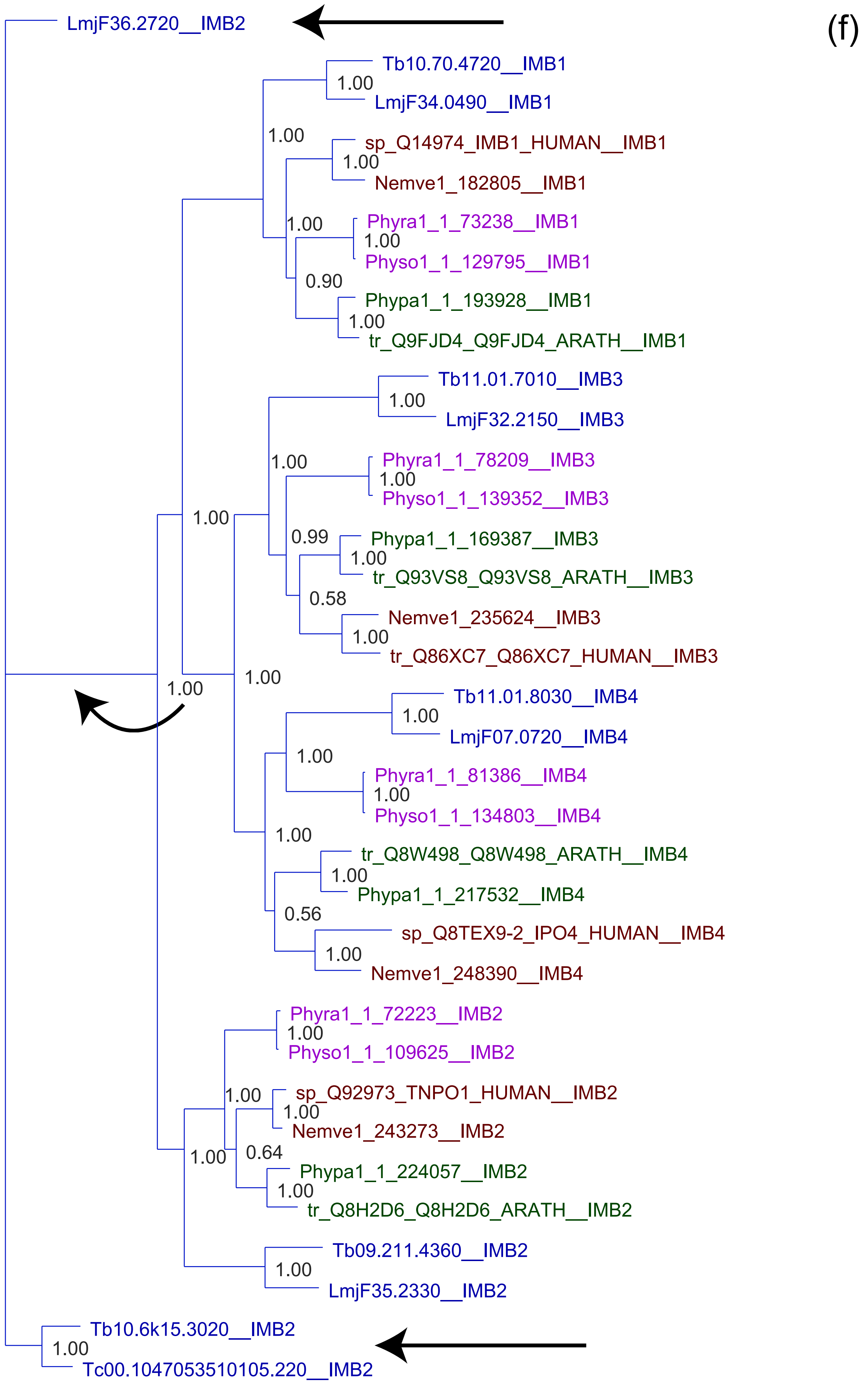
(f)
LmjF36.2720__IMB2
Tb10.70.4720__IMB1
1.00
LmjF34.0490__IMB1
1.00
sp_Q14974_IMB1_HUMAN__IMB1
1.00
Nemve1_182805__IMB1
1.00
Phyra1_1_73238__IMB1
1.00
Physo1_1_129795__IMB1
0.90
Phypa1_1_193928__IMB1
1.00
tr_Q9FJD4_Q9FJD4_ARATH__IMB1
Tb11.01.7010__IMB3
1.00
LmjF32.2150__IMB3
1.00
Phyra1_1_78209__IMB3
1.00
Physo1_1_139352__IMB3
1.00
0.99
Phypa1_1_169387__IMB3
1.00
tr_Q93VS8_Q93VS8_ARATH__IMB3
0.58
Nemve1_235624__IMB3
1.00
tr_Q86XC7_Q86XC7_HUMAN__IMB3
1.00
1.00
Tb11.01.8030__IMB4
1.00
LmjF07.0720__IMB4
1.00
Phyra1_1_81386__IMB4
1.00
Physo1_1_134803__IMB4
1.00
tr_Q8W498_Q8W498_ARATH__IMB4
1.00
Phypa1_1_217532__IMB4
0.56
sp_Q8TEX9-2_IPO4_HUMAN__IMB4
1.00
Nemve1_248390__IMB4
Phyra1_1_72223__IMB2
1.00
Physo1_1_109625__IMB2
1.00
sp_Q92973_TNPO1_HUMAN__IMB2
1.00
Nemve1_243273__IMB2
1.00
0.64
Phypa1_1_224057__IMB2
1.00
tr_Q8H2D6_Q8H2D6_ARATH__IMB2
Tb09.211.4360__IMB2
1.00
LmjF35.2330__IMB2
Tb10.6k15.3020__IMB2
1.00
Tc00.1047053510105.220__IMB2

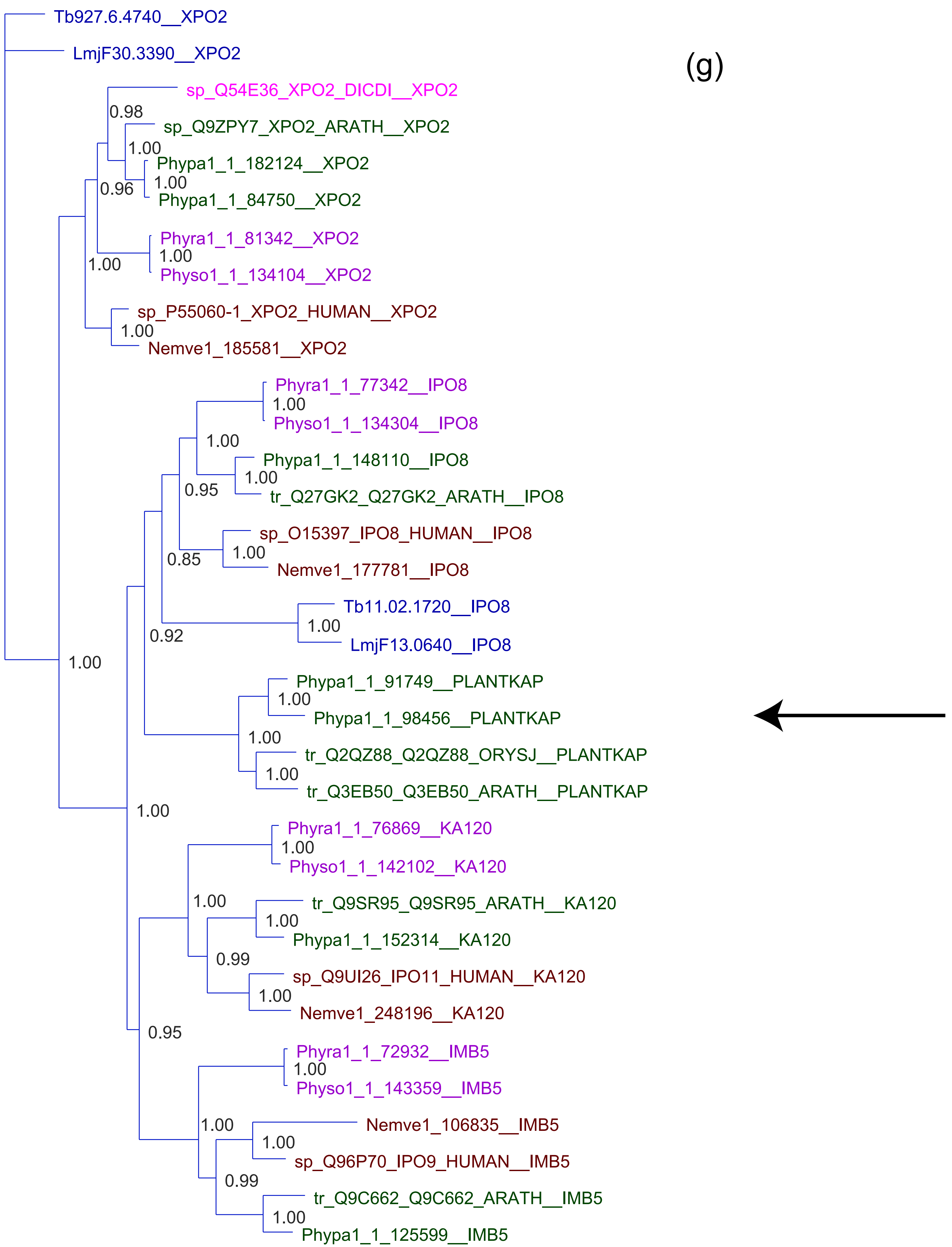
(g)
Tb927.6.4740__XPO2
LmjF30.3390__XPO2
sp_Q54E36_XPO2_DICDI__XPO2
0.98
sp_Q9ZPY7_XPO2_ARATH__XPO2
1.00
Phypa1_1_182124__XPO2
0.96
1.00
Phypa1_1_84750__XPO2
Phyra1_1_81342__XPO2
1.00
1.00
Physo1_1_134104__XPO2
sp_P55060-1_XPO2_HUMAN__XPO2
1.00
Nemve1_185581__XPO2
Phyra1_1_77342__IPO8
1.00
Physo1_1_134304__IPO8
1.00
Phypa1_1_148110__IPO8
1.00
0.95
tr_Q27GK2_Q27GK2_ARATH__IPO8
sp_O15397_IPO8_HUMAN__IPO8
1.00
0.85
Nemve1_177781__IPO8
Tb11.02.1720__IPO8
1.00
0.92
LmjF13.0640__IPO8
1.00
Phypa1_1_91749__PLANTKAP
1.00
Phypa1_1_98456__PLANTKAP
1.00
tr_Q2QZ88_Q2QZ88_ORYSJ__PLANTKAP
1.00
tr_Q3EB50_Q3EB50_ARATH__PLANTKAP
1.00
Phyra1_1_76869__KA120
1.00
Physo1_1_142102__KA120
1.00
tr_Q9SR95_Q9SR95_ARATH__KA120
1.00
Phypa1_1_152314__KA120
0.99
sp_Q9UI26_IPO11_HUMAN__KA120
1.00
Nemve1_248196__KA120
0.95
Phyra1_1_72932__IMB5
1.00
Physo1_1_143359__IMB5
1.00
Nemve1_106835__IMB5
1.00
sp_Q96P70_IPO9_HUMAN__IMB5
0.99
tr_Q9C662_Q9C662_ARATH__IMB5
1.00
Phypa1_1_125599__IMB5

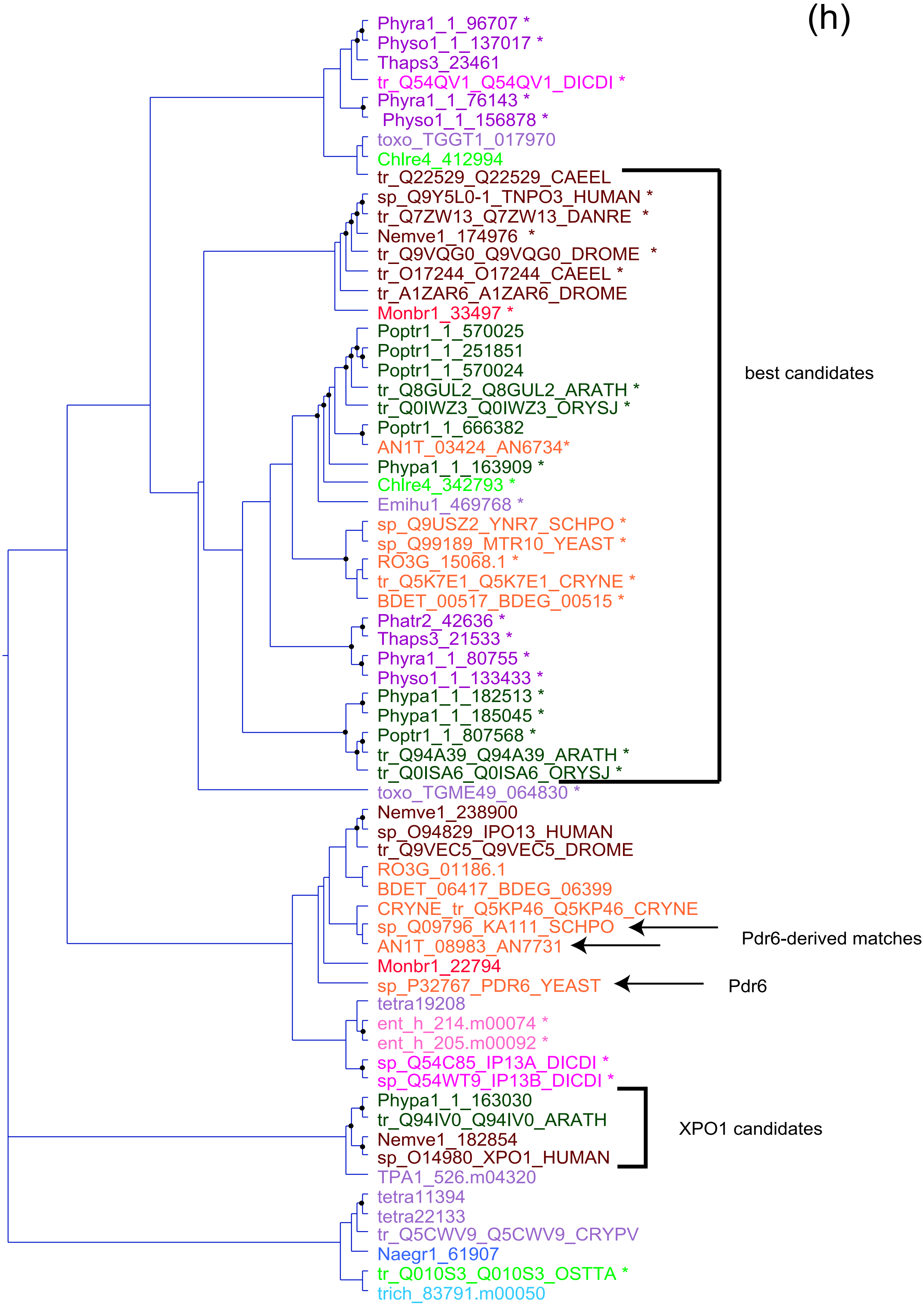

ClustalW-generated NJ cladogram of all TNPO3 subfamily candidates and a small number of XPO1 subfamily candidates. The cluster of best TNPO3 candidates, the XPO1 clade, and matches detected by Pdr6 are all indicated. All TNPO3 candidates that verified as subfamily members by phylogeny are marked with an asterisk. Nodes with bootstrap support greater than 90% are marked with a dot.
